# Deep phenotyping of oxidative stress in emergency room patients reveals homoarginine as a novel predictor of sepsis severity, length of hospital stay, and length of intensive care unit stay

**DOI:** 10.3389/fmed.2022.1033083

**Published:** 2022-11-25

**Authors:** Mei Li Ng, Win Sen Kuan, Leroy Sivappiragasam Pakkiri, Eugene Chen Howe Goh, Lik Hang Wu, Chester Lee Drum

**Affiliations:** ^1^Department of Medicine, Yong Loo Lin School of Medicine, National University of Singapore, Singapore, Singapore; ^2^Emergency Medicine Department, National University Hospital, National University Health System, Singapore, Singapore; ^3^Cardiovascular Research Institute, National University Health System, Singapore, Singapore; ^4^Department of Biochemistry, Yong Loo Lin School of Medicine, National University of Singapore, Singapore, Singapore

**Keywords:** homoarginine, methylarginines, sepsis, critical care, emergency medicine

## Abstract

**Background:**

We aimed to determine primary markers of oxidative stress (OS) in ED patients which predict hospital length of stay (LoS), intensive care unit (ICU) LoS, and sepsis severity.

**Materials and methods:**

This prospective, single center observational study was conducted in adult patients recruited from the ED who were diagnosed with either sepsis, infection without sepsis, or non-infectious, age-matched controls. 290 patients were admitted to the hospital and 24 patients had direct admission to the ICU. A panel of 269 OS and related metabolic markers were profiled for each cohort. Clinical outcomes were direct ICU admission, hospital LoS, ICU LoS, and *post-hoc*, adjudicated sepsis severity scoring. Bonferroni correction was used for pairwise comparisons. Principal component regression was used for dimensionality reduction and selection of plasma metabolites associated with sepsis. Multivariable negative binomial regression was applied to predict admission, hospital, and ICU LoS.

**Results:**

Homoarginine (hArg) was the top discriminator of sepsis severity [sepsis vs. control: ROC-AUC = 0.86 (95% CI 0.81–0.91)], [sepsis vs. infection: ROC-AUC = 0.73 (95% CI 0.68–0.78)]. The 25th percentile of hArg [odds ratio (OR) = 8.57 (95% CI 1.05–70.06)] was associated with hospital LoS [IRR = 2.54 (95% CI 1.83–3.52)] and ICU LOS [IRR = 18.73 (95% CI 4.32–81.27)]. In prediction of outcomes, hArg had superior performance compared to arginine (Arg) [hArg ROC-AUC = 0.77 (95% CI 0.67–0.88) vs. Arg ROC-AUC = 0.66 (95% CI 0.55–0.78)], and dimethylarginines [SDMA ROC-AUC 0.68 (95% CI 0.55–0.79) and ADMA ROC-AUC = 0.68 (95% CI 0.56–0.79)]. Ratio of hArg and Arg/NO metabolic markers and creatinine clearance provided modest improvements in clinical prediction.

**Conclusion:**

Homoarginine is associated with sepsis severity and predicts hospital and ICU LoS, making it a useful biomarker in guiding treatment decisions for ED patients.

## Introduction

Sepsis is a dysregulated response to infection that predisposes to multiple organ failure and death. It is a serious global health crisis affecting 48.9 million people and there are an estimated 11 million sepsis-related deaths every year, representing 20% of global mortality (1). Identifying sepsis in the emergency department (ED) is a critical challenge, with every hour delay in treatment increasing the risk of mortality by 10% (2). Sepsis is characterized by the production of oxidative stress (OS) which originates from multiple biological sources. While regulated production of reactive oxygen species (ROS) produced by localized inflammation may be adaptive, the release of ROS in unregulated systemic inflammation and mitochondrial dysfunction can lead to end organ damage and loss of vascular tone (3).

Although free radicals have been quantified in sepsis patients using electron paramagnetic resonance (4), quantifying OS in a clinical setting remains a challenge given the short half-lives of ROS, which are often measured in seconds. Scalable techniques for quantification of OS in clinical cohorts must, therefore, rely on indirect measures of OS and markers of OS mediated damage. We divide OS measures into three general classes, (1) lipid and metabolite oxidation products, (2) OS buffering systems and antioxidants, and (3) markers of energy metabolism and immune system activity. Markers from the first class, i.e., F2-isoprostanes and isofurans, are associated with multisystem organ failure (MOF), however, do not correlate with severity of sepsis or clinical outcomes (5). A cumulative measure of lipid peroxidation, i.e., thiobarbituric acid reactive substances (TBARS), is associated with MOF and sequential organ failure assessment (SOFA) scores in patients admitted to the intensive care unit (ICU) (6). Markers in the second class, i.e., measures of glutathione, glutathione redox ratio (GSSG/GSH), solvent accessible thiols, and glutathione peroxidase activity, are associated with sepsis severity in pediatric sepsis (6–9). Lastly, markers from the third class, i.e., xanthine metabolites, (a downstream product of ATP production), and xanthine oxidase (XO) activity are predictors of sepsis severity and correlates with markers from both class 1 (lipid peroxidation) and class 2 (GSSG/GSH) (9). Nitric oxide (NO) is a paracrine regulator of vascular tone, and is also a member of class 3 as it is produced by the immune system to produce reactive nitrogen species (RNS), i.e., peroxynitrite, which directly damage target proteins and contribute to vascular dysregulation (10). Both the physiologic substrate of nitric oxide synthase (NOS), arginine (Arg), and physiologic inhibitors of NOS, asymmetric dimethylarginine (ADMA) and symmetric dimethylarginine (SDMA), correlate with sepsis severity, and clinical outcomes; however, clinically relevant discrimination of sepsis from general infection and prediction of hospital LoS has not been demonstrated. We hypothesized that deep phenotyping of OS marker(s) could more precisely predict clinical outcomes in patients with sepsis.

Herein, we used OS “deep phenotyping” approach to characterize multiple categories of OS markers, including lipid peroxidation, thiols, purine metabolites, and nitrosative stress. We combined the measurement of 269 OS-related markers with immunoassay and clinical data to phenotype ED patients suspected of having infection or sepsis and evaluated the association between OS markers and clinical outcomes.

## Materials and methods

### Study design, setting, and patient enrolment

This prospective, single-center observational study enrolled patients who presented with infection and sepsis at the ED of National University Hospital in Singapore under ethics approval (reference number 2013/00554). The study was registered with ClinicalTrials.gov (NCT02544490). Between September 2013 and November 2018, adult patients in the ED with suspected or confirmed infection were screened and approached for informed consent and subsequent participation into the study. Patients without suspected or confirmed infection were recruited as controls. Exclusion criteria were age below 21 years, known pregnancy, prisoners, do-not-attempt resuscitation status, requirement for immediate surgery, active chemotherapy, hematological malignancy, treating physician deems aggressive care unsuitable, those unable to give informed consent, and unable to comply with study requirements. Demographic, clinical, and laboratory measurements were recorded prospectively. Positive cultures (from blood, urine, endotracheal tube, sputum) were used to confirm presence of infection. Decisions related to ICU or hospital admission were made by the treating ED physician. Assessment of organ failure or deterioration at the ED presentation was evaluated by SOFA score. Patients requiring intensive care were typically admitted to the ICU within 4–8 h of their ED arrival. Patients were categorized into three groups: (1) admitted directly to the ICU from the ED; (2) admitted to ward from the ED; and (3) discharged from the ED. A detailed description of data and blood samples collection are provided in the Supplementary methods. To ensure the predictive accuracy of plasma metabolites identified at the earliest ED presentation, patients who were discharged directly from the ED were followed up for any clinical recurrence of their condition within 28 days.

### Sampling timepoint

The first sampling for the study was done in conjunction with the initial clinical laboratory blood draw for all subjects, prior to any disposition plans. Those who were subsequently admitted to the general ward or ICU had their 2nd (24–48 h) and 3rd blood samples taken (48–72 h) post admission. Plasma was extracted from whole blood samples and stored in the Tissue Repository pending analysis. The 1st blood sample was used for metabolomic profiling study (Supplementary Figure 1), and the identification of prognostic and predictive markers of the metabolomic panel.

### Outcome measures

The primary outcome was plasma metabolite(s) level and/or ratio associated with sepsis severity and length of stay (LoS). LoS was defined as the total number of days each patient stay in the hospital (hospital LoS) or ICU (ICU LoS). Any amount of time spent during a 24 h period was recorded as one full day.

### Statistical analysis

Continuous variables are presented as mean ± SD and interquartile ranges (interquartile range), normality is checked by the Shapiro–Wilk test. Categorical variables are reported as frequency (percentage). Ethnicity and predisposing factors were dummy coded for each independent *t*-test. Baseline characteristics of each group were compared using analysis of variance (ANOVA) and a Bonferroni *post-hoc* test for continuous variables, or χ^2^ test for categorical variables, at an adjusted significance level of *P* < 0.05. Metabolomics data were normalized in three steps: (i) samples normalization to the total of all obtained values as a general-purpose correction for sample differences; (ii) data transformation using log base 2-transformation; and (iii) data scaling using an auto scaling algorithm. Description of group differences was initially a univariate analysis with parametric testing and Benjamini–Hochberg correction to control for experiment wise error rate (α = 0.05), then visualized as a volcano plot (11). An unsupervised principal component analysis (PCA) was performed for multivariate analysis to reduce the dimension of metabolite variables and detect outliers (sample data > 95% confidence interval [CI] of the Hotelling’s T2 distribution) (12). Based on the pre-processed original data, and following supervised PLS-DA, metabolites in the first two principal components (PC-1, PC-2) were weighted by the variable importance in projection (VIP) score, with cross validation (CV) applied to maximize the correlation between *X* matrices of metabolites and *Y* outcomes. In the supervised analysis, the VIP score of a variable was calculated as weighted sum of the squared correlations between the PLS-DA components and the original variable, which indicates the importance of the variable to the class discrimination. Variables with VIP score > 2 was selected as significant metabolites. Finally, the most significant metabolites related to sepsis were identified using sparse partial least squares discriminant analysis (sPLS-DA), which the degree of sparsity (i.e., the equivalent number of selected metabolites) in each PLS-DA component was optimized using LASSO penalized regression algorithm (13). To assess model fit and avoid overfitting, model fit was validated using three performance indicators: (1) A 10-fold CV (i.e., R2Y and Q2 metrics), (2) Permutation tests (i.e., empirical *P*-value < 5*e*−04 was used in 2,000 permutation test), and (3) Receiver operating characteristic (ROC) (14). Spearman’s rank correlation was used to assess pairwise correlations. We applied plasma metabolite data, demographic data and clinical data to develop optimal regression models for prognosis, at which potential confounders including age, gender, and comorbidities (hypertension, hyperlipidaemia, cardiovascular disease, and cancer) were considered, and significant variables were chosen by a stepwise selection method (Hosmer and Lemeshow goodness-of-fit test *P* > 0.05) (15). Odd ratio (OR) comparing the stratified percentiles: low percentile (25th percentile), intermediate percentile (25th–75th percentile), and high percentile (75th percentile), of plasma metabolite values and ratios, the 95% CIs, and the corresponding *P*-values, to evaluate additive values of the OS biomarkers (with and without clinical variables) for outcomes in the logistic regression model (16). A receiver operating characteristic (ROC) curve with area under the curve (AUC) was used to evaluate the predictive performance of each regression model. Negative binomial regression was used to model the relationship between metabolites and LoS, which incidence rate ratio (IRR), 95% CI, and *P*-value from the likelihood ratio test comparing different percentile range were performed (17). Statistical significance was two-sided at α = 0.05. Statistical analyses were performed using SPSS (version 26; IBM Corporation, Armonk, NY, USA) and open-source R packages (version 4.1.2). Multivariate regression was performed using MetaboAnalyst (version 4.0) (18).

## Results

### Patient characteristics

**FIGURE 1:**
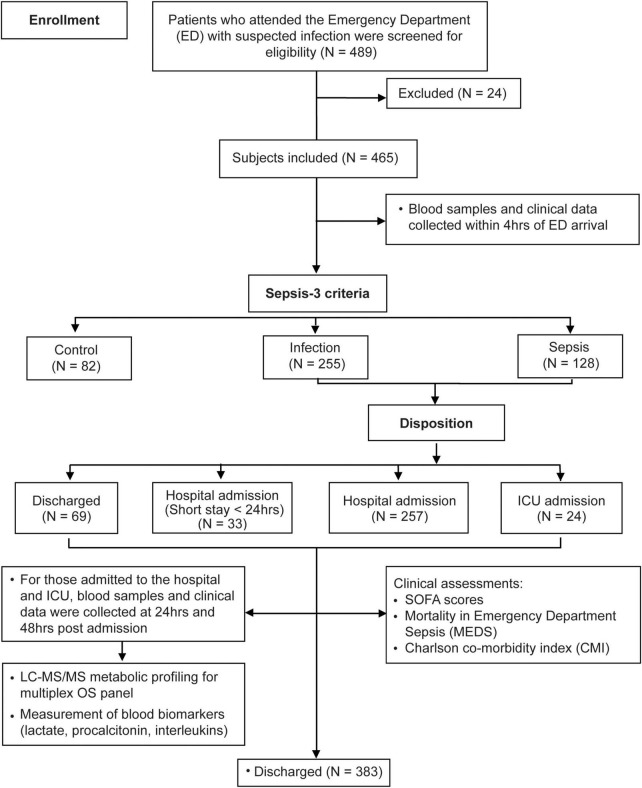
Study design and activities.

Under the study design, 489 patients were enrolled of whom 465 met study criteria (Figure 1). Subjects were classified into controls (*N* = 82), infection (*N* = 255), and sepsis (*N* = 128) using Sepsis-3 criteria, baseline characteristics are shown in Table 1 with further detail in Supplementary Table 1. Overall, 37 sepsis patients had a deterioration of SOFA, with 64.90% (*N* = 24) admitted to hospital ward and 35.10% (*N* = 13) admitted to ICU. Compared to patients with infection, those with sepsis had longer hospital LoS (3.27 ± 4.20 vs. 7.93 ± 8.75 days) and ICU LoS (0.04 ± 0.38 vs. 1.16 ± 3.49), *P* < 0.001.

**TABLE 1:** Baseline characteristics of patients at emergency department (ED) admission.

Baseline characteristics	Control	Infection	Sepsis	*P*-value^a^
	
	(*N* = 82)	(*N* = 255)	(*N* = 128)	
Age (years)	48.94 ± 11.03	48.94 ± 14.93	57.74 ± 13.21	<0.001
Gender, *n* (%)				
Male	49 (59.80)	143 (54.60)	85 (66.40)	0.026
Female	33 (40.20)	119 (45.40)	43 (33.60)	
BMI	26.32 ± 4.90	27.86 ± 6.72	26.91 ± 5.82	0.221
Temperature (^°^C)	36.70 ± 0.40	38.52 ± 0.96	38.65 ± 0.97	0.206
Heart rate (min^–1^)	80.16 ± 12.43	105.11 ± 16.60	107.98 ± 19.69	0.133
Systolic blood pressure (mmHg)	n.a	130.78 ± 22.64	126.16 ± 25.41	0.070
Diastolic blood pressure (mmHg)	n.a	76.36 ± 11.13	72.05 ± 13.24	0.001
Mean arterial pressure (mmHg)	n.a	94.49 ± 13.54	90.08 ± 15.85	0.005
Respiratory rate (min^–1^)	n.a	20.08 ± 3.80	20.61 ± 4.58	0.230
WBC counts (×10^9^/L)	7.48 ± 1.73	12.33 ± 5.66	13.17 ± 13.03	0.381
RBC counts (×10^12^/L)	4.85 ± 0.46	4.71 ± 0.54	4.46 ± 0.83	<0.001
Haematocrit (%)	41.33 ± 3.57	39.59 ± 4.37	37.68 ± 6.04	<0.001
Platelet count (×10^9^/L)	274.03 ± 61.21	261.5 ± 86.59	200.99 ± 98.24	<0.001
Serum lactate (mmol/L)	Na	1.86 ± 1.64	1.94 ± 1.02	0.713
C-reactive protein (mg/L)	7.29 ± 4.72	91.97 ± 85.99	109.61 ± 94.39	0.078
Procalcitonin (μg/L)	0.16 ± 0.55	1.71 ± 6.41	10.49 ± 31.97	<0.001
Interleukin-6 (pg/ml)	5.15 ± 1.83	48.70 ± 54.33	2442.36 ± 2336.11	0.028
Interleukin-8 (pg/ml)	3.45 ± 2.90	2.00 ± 0	289.58+384.54	0.001
**Comorbidities**				
Hypertension	31 (37.80)	99 (37.80)	80 (62.50)	<0.001
Dyslipidaemia	32 (39)	94 (35.90)	73 (57)	<0.001
Cardiovascular disease	8 (9.80)	38 (14.50)	36 (28.10)	0.001
Cancer	0	6 (2.30)	8 (6.30)	0.048
**Disposition, *n* (%)**				
Discharge	n.a	58 (22.10)	5 (3.90)	<0.001
General ward	n.a	176 (67.20)	93 (72.70)	0.272
Ward admission	n.a	25 (9.50)	9 (7)	0.409
ICU admission	n.a	3(1.10)	21(16.40)	<0.001
Hospital LoS (days)	n.a	3.27 ± 4.20	7.93 ± 8.75	<0.001
ICU LoS (days)	n.a	0.04 ± 0.38	1.16 ± 3.49	<0.001

Data presented: means ± SD and n (%). ^a^*P*-value: analysis of variance (ANOVA) and Bonferroni post-hoc, or χ^2^ test. *P* < 0.05 is statistically significant. ED, emergency department; WBC, white blood cells; RBC, red blood cells; IPTT, activated partial thromboplastin time; INR, initial international normalized ratio; MEDS, mortality in emergency department sepsis; SOFA, sequential organ failure assessment; QSOFA, quick sequential organ failure assessment; LoS, length of stay. n.a, data not available.

The univariate (volcano) and multivariate (PCA, PLS-DA) identified five major metabolites associated with sepsis severity – these were lysine, glutamine, homoarginine (hArg), arginine (4 NO substrates), and SDMA (inhibitor of arginine transport). The results of the analysis framework (Supplementary Figure 1) and identification of the five key markers are detailed as Supplementary Figures 3, 4.

### Biomarkers for diagnosis

**FIGURE 2:**
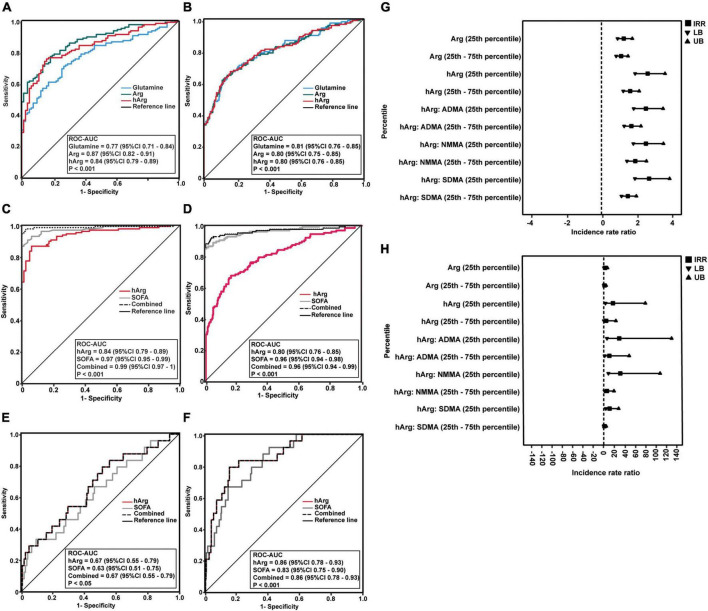
Predictability of homoarginine (hArg) for sepsis and clinical outcomes. **(A–D)** Receiver operator characteristic (ROC) curves of glutamine (blue line), arginine (Arg) (green line), and hArg (red line) for sepsis diagnosis. **(A,C)** ROC of the predicted probability score for sepsis vs. control, without **(A)** or with sequential organ failure assessment (SOFA) **(C)**. **(B,D)** ROC curve of the predicted probability score for sepsis vs. infection, without **(B)** or with SOFA **(D)**. ROC curves were adjusted for age, creatinine, and co-morbidities. **(E)** ROC curves of SOFA (gray line), hArg (red line), and combined (SOFA + hArg) (butted line) for hospital admission. **(F)** ROC curves of SOFA (gray line), hArg (red line), and combined (SOFA + hArg) (butted line) for intensive care unit (ICU) admission. ROC curves were adjusted for age, creatinine, and comorbidities. **(G)** Forest plot shows a negative binomial (log link) model and incidence rate ratio for comparison of hospital length of stay (LoS) (days) in patients with sepsis (*N* = 128) and infection (*N* = 255). *^a^P*-value < 0.05 is statistically significant for hospital LoS (days). **(H)** Negative binomial (log link) model and incidence rate ratio for ICU LoS (days) in patients with sepsis (*N* = 128) and infection (*N* = 255). *^b^P*-value < 0.05 is statistically significant. Each model predicts LoS stratified by low percentile (25th percentile) and intermediate percentile (25th–75th percentile) of Arg and hArg at admission. Each model was adjusted for age, gender, creatinine, and comorbidities (hypertension, hyperlipidaemia, cardiovascular disease, and cancers).

The ability of glutamine, Arg, and hArg to diagnose sepsis was assessed using multivariate logistic regression adjusted for age, gender, baseline serum creatinine, and co-morbidities, as these variables were significantly different in the univariate analysis (Supplementary Figures 3, 4). In sepsis vs. control groups, the ROC diagnostic model achieved glutamine ROC-AUC of 0.77 (95% CI 0.71–0.84), Arg ROC-AUC of 0.87 (95% CI 0.82–0.91), and hArg ROC-AUC of 0.84 (95% CI 0.79–0.89), *P* < 0.001 (Figure 2A). These biomarkers performed nearly equally well in the sepsis vs. infection groups (Figure 2B).

Following that, glutamine was eliminated because it had the lowest ROC-AUC of 0.77 (95% CI = 0.71–0.84) when compared to Arg and hArg (Figure 2A). Combining hArg with SOFA score resulted in the best diagnostic performance, with ROC-AUC of 0.99 (95% CI 0.97–1) (Figure 2C) and ROC-AUC of 0.96 (95% CI 0.94–0.99), *P* < 0.001 (Figure 2D). Overall, the results indicate that hArg’s potential role in sepsis diagnosis.

### Prognostic prediction of homoarginine for admission

Diastolic arterial pressure (DAP) is a marker of exacerbated vasodilation (19, 20). To examine its relationship with hArg, we stratified hArg plasma percentiles as low (25th percentile), intermediate (25th–75th percentile), and high (75th percentile), and build multivariable linear regression (LR) models for DAP (Supplementary Table 6), admission, and LoS, respectively. Multivariable LR models comparing Arg, hArg and methylarginines, the low (25th) percentile hArg was strongly predictive for hospital [odds ratio (OR) = 2.71 (95% CI 1.16–6.30), *P* = 0.021] and ICU admission [OR = 8.57 (95% CI 1.05–70.06), *P* = 0.045] (Table 2). The predictability of each admission was further improved by considering the ratio of hArg to dimethylarginines, which had the highest predictive values for ICU admission with low (25th) percentile hArg:ADMA [OR = 10.16 (95% CI 1.36–91.03), *P* = 0.024], hArg:SDMA [OR = 5.29 (95% CI 1.28–21.72), *P* = 0.021], and hArg: N-monomethylarginine (NMMA) [OR = 10.44 (95% CI 1.67–65.25), *P* = 0.012]. Arg was not associated with hospital or ICU admission. Figures 2E,F depicts the performance of hArg for hospital [ROC-AUC of 0.67 (95% CI 0.55–0.79), *P* < 0.05] and ICU admission [ROC-AUC of 0.86 (95% CI 0.78–0.93), *P* < 0.001]. Supplementary Table 7 summarizes the *Z*-score for pairwise ROC curves comparison. The *Z* score compared two ROC-AUC values for each pairwise model, and the predictive performance for ICU admission was significantly improved with the combined model [SOFA + metabolite model] [*Z* = −2.632, ROC-AUC difference = −0.08 (95% CI -0.14–0.02), *P* = 0.008].

**TABLE 2:** Independent metabolic predictors of hospital and intensive care unit (ICU) admission.

Sepsis cohort	Hospital (*N* = 290)				ICU (*N* = 24)			
		
	Univariable		Multivariable		Univariable		Multivariable	
				
	OR [95% CI]^a^	*P*-value^a^	OR [95% CI]^b^	*P*-value^b^	OR [95% CI]^a^	*P*-value^a^	OR [95% CI]^b^	*P*-value^b^
**Arg**								
25th percentile	1.61 [0.75–3.42]	0.216	1.77 [0.75–4.15]	0.185	1.30 [0.38–4.42]	0.667	1.57 [0.44–5.56]	0.484
25th–75th percentile	0.76 [0.40–1.45]	0.413	0.89 [0.43–1.82]	0.751	0.87 [0.27–2.86]	0.829	0.94 [0.28–3.16]	0.370
**hArg**								
25th percentile	2.93 [1.36–6.31]	0.006	2.71 [1.16–6.30]	0.021	8.66 [1.03–63.12]	0.039	8.57 [1.05–70.06]	0.045
25th–75th percentile	1.43 [0.76–2.69]	0.255	1.75 [0.87–3.50]	0.113	2.19 [0.26–17.93]	0.003	2.04 [0.24–17.12]	0.032
**ADMA**								
75th percentile	1.45 [0.73–2.87]	0.882	0.85 [0.43–1.68]	0.659	1.52 [0.63–3.67]	0.350	1.23 [0.48–3.16]	0.654
25th–75th percentile	1.04 [0.59–1.82]	0.283	0.98 [0.45–2.10]	0.958	0.69 [0.30–1.61]	0.401	0.73 [0.31–1.74]	0.486
**SDMA**								
75th percentile	5.11 [2.01–12.97]	<0.001	1.36 [0.45–4.09]	0.582	2.92 [1.26–6.73]	0.012	2.30 [0.80–6.60]	0.119
25th–75th percentile	1.67 [0.08–3.41]	0.156	1.12 [0.51–2.47]	0.765	0.44 [0.17–1.09]	0.078	0.55 [0.21–1.45]	0.229
**NMMA**								
75th percentile	1.38 [0.70–2.74]	0.988	0.82 [0.38–1.76]	0.622	1.50 [0.62–3.63]	0.363	1.35 [0.53–3.40]	0.523
25th–75th percentile	1.00 [0.57–1.75]	0.344	0.73 [0.39–1.36]	0.328	0.85 [0.37–1.95]	0.703	0.79 [0.34–1.86]	0.599
**hArg: ADMA**								
25th percentile	4.93 [2.32–10.43]	<0.001	3.99 [1.73–9.21]	0.001	13.28 [1.69–104.36]	0.014	10.16 [1.36–91.03]	0.024
25th–75th percentile	2.30 [1.36–3.89]	0.002	2.34 [1.30–4.20]	0.004	5.74 [0.73–45.20]	0.097	5.09 [0.64–40.58]	0.124
**hArg: SDMA**								
25th percentile	7.46 [3.11–17.92]	<0.001	4.20 [1.48–11.92]	0.007	5.28 [1.48–18.89]	0.010	5.29 [1.28–21.72]	0.021
25th–75th percentile	1.92 [1.14–3.22]	0.014	1.77 [1.00–3.14]	0.050	1.03 [0.25–4.22]	0.963	0.99 [0.24–41.23]	0.993
**hArg: NMMA**								
25th percentile	4 [1.98–8.06]	<0.001	3.02 [1.36–6.69]	0.006	13.42 [2.24–80.25]	0.004	10.44 [1.67–65.25]	0.012
25th–75th percentile	3.14 [1.83–5.37]	<0.001	3.51 [1.92–6.43]	<0.001	3.39 [0.77–14.92]	0.105	2.77 [0.61–12.54]	0.185

Data presented: OR [95% CI]. Plasma presented: 25th percentile, 25th–75th percentile, and 75th percentile. Reference group (75th percentile) for Arg, hArg, and ratios (hArg: ADMA, hArg: SDMA, and hArg: NMMA). Reference group (25th percentile) for methylarginines (ADMA, SDMA, NMMA), respectively. OR [95% CI]^a^ and *P*-value^a^ by univariable logistic regression, OR [95% CI]^b^ and *P*-value^b^ by multivariable logistic regression adjusted for age, gender, creatinine, and comorbidities. *P* < 0.05 is statistically significant. OR, odd ratio; 95% CI, 95% confidence interval.

### Prognostic prediction of homoarginine for length of stay

Intensive care unit LoS is a critical clinical outcome that influences hospital costs, risks of in-hospital complications and long-term morbidity. To overcome overdispersion for count data (LoS), we used a Bayesian estimate of negative binomial (BN) model to predict the LoS of those patients with low (25th) percentile of hArg, controlling for other confounders. The BN model was tested for goodness of fit [deviance (value/df) = 0.97, Akaike’s Information Criterion (AIC) = 515.66], and Likelihood Ratio (χ^2^ = 155.14, *P* < 0.001). Mean (SD) hospital LoS and ICU LoS were 4.90 (6.48) days and 0.41 (2.08) days, respectively. Patients were stratified into two groups based on their plasma hArg percentiles: low (25th) and intermediate (25th–75th). Dimethylarginines or Arg were used as comparators. Forest plots (Figures 2G,H) showed the risk estimate, for hospital and ICU LoS. The IRR calculated the number of incidence event per number of patient-days. The univariable (unadjusted), and multivariable (adjusted) BN models are summarized in Table 3. Arg was not a significant predictor of hospital LoS. hArg [IRR = 2.54 (95% CI 1.83–3.52), *P* < 0.001] and hArg to methylarginines’ ratios were significant predictors of hospital LoS (reference group = 75th percentile). The multivariable model estimated hospital LoS for a patient with low (25th) percentile was 7.17 days, and for those with intermediate (25th–75th) percentile was 6.08 days (reference group = 75th percentile). For ICU LoS, hArg [IRR = 18.73 (95% CI 4.32–81.27), *P* < 0.001] and hArg to methylarginines’ ratios were significant predictors, of which 5.25 times higher risk compared to Arg [IRR = 3.57 (95% CI 1.61–7.93), *P* = 0.002]. The model’s estimated ICU LoS for a patient with low (25th) percentile was 21.40 days, and with intermediate (25th–75th) percentile was 13.48 days (reference group = 75th percentile). Overall, the results point to a potentially superior role for hArg in risk stratification of ED patients with infection or sepsis.

**TABLE 3:** Incidence rate ratio for hospital and intensive care unit (ICU) length of stay (LoS) stratified by percentiles of homoarginine (hArg), asymmetric dimethylarginine (ADMA), symmetric dimethylarginine (SDMA), and N-monomethylarginine (NMMA).

	Hospital (*N* = 290) LoS				ICU (*N* = 24) LoS			
		
	IRR [95% CI]^a^	*P*-value^a^	IRR [95% CI]^b^	*P*-value^b^	IRR [95% CI]^a^	*P*-value^a^	IRR [95% CI]^b^	*P*-value^b^
**Arg**								
25th percentile	1.26 [0.90–1.75]	0.177	1.19 [0.84–1.67]	0.330	3.72 [1.79–7.73]	<0.001	3.57 [1.61–7.93]	0.002
25th–75th percentile	1.08 [0.79–1.47]	0.631	1.04 [0.76–1.43]	0.804	2.61 [1.28–5.33]	0.008	2.50 [1.16–5.39]	0.020
**hArg**								
25th percentile	2.55 [1.78–3.65]	<0.001	2.54 [1.83–3.52]	<0.001	25.47 [6.06–107.01]	<0.001	18.73 [4.32–81.27]	<0.001
25th–75th percentile	1.35 [0.97–1.89]	0.078	1.55 [1.16–2.06]	0.003	7.13 [1.69–30.09]	0.008	5.59 [1.28–24.39]	0.022
**ADMA**								
75th percentile	1.09 [0.17–6.71]	0.920	0.37 [0.06–2.22]	0.279	1.32 [0.74–2.37]	0.351	1.51 [0.76–2.97]	0.237
25th–75th percentile	0.46 [0.09–2.20]	0.331	0.33 [0.07–1.53]	0.159	0.97 [0.59–1.61]	0.910	0.90 [0.49–1.64]	0.728
**NMMA**								
75th percentile	4.10 [0.67–25.07]	0.126	1.21 [0.20–7.35]	0.833	1.45 [0.81–2.60]	0.213	1.85 [0.94–3.66]	0.076
25th–75th percentile	1.51 [0.31–7.24]	0.602	0.73 [0.15–3.41]	0.693	0.95 [0.57–1.57]	0.832	0.59 [0.31–1.14]	0.116
**SDMA**								
75th percentile	2.48 [1.83–3.37]	<0.001	17.84 [2.46–129.19]	0.004	2.17 [1.24–.82]	0.007	6.03 [2.63–13.85]	<0.001
25th–75th percentile	1.37 [1.04–1.80]	0.025	2.61 [0.57–11.81]	0.213	1.14 [0.69–1.88]	0.601	2.63 [1.22–5.70]	0.014
**hArg: ADMA**								
25th percentile	2.80 [2.04–3.84]	<0.001	2.44 [1.75–3.41]	<0.001	45.03 [10.79–188.02]	<0.001	30.84 [7.20–132.03]	<0.001
25th–75th percentile	1.65 [1.24–2.18]	0.001	1.62 [1.21–2.16]	0.001	16 [3.84–66.73]	<0.001	11.77 [2.77–50.05]	0.001
**hArg: NMMA**								
25th percentile	4.50 [2.63–7.68]	<0.001	2.44 [1.73–3.43]	<0.001	39.47 [12.38–125.91]	<0.001	32.97 [9.91–109.74]	<0.001
25th–75th percentile	1.99 [1.53–2.60]	<0.001	1.84 [1.37–2.47]	<0.001	11.18 [4.02–31.06]	<0.001	7.24 [2.54–20.66]	<0.001
**hArg: SDMA**								
25th percentile	3.13 [2.30–4.26]	<0.001	2.62 [1.82–3.78]	<0.001	16.04 [7.13–36.09]	<0.001	12.27 [5.07–29.69]	<0.001
25th–75th percentile	1.43 [1.08–1.89]	0.014	1.42 [1.06–1.90]	0.020	2.44 [1.04–5.73]	0.040	2.38 [1–5.66]	0.051

Data presented: IRR [95% confidence interval]. Plasma presented: 25th percentile, 25th–75th percentile, and 75th percentile. Reference group (75th percentile) for Arg, hArg, and ratios (hArg: ADMA, hArg: SDMA, and hArg: NMMA). Reference group (25th percentile) for methylarginines (ADMA, SDMA, NMMA), respectively. IRR [95% CI]^a^ and *P*-value^a^ by univariable negative binomial regression, IRR [95% CI]^b^ and *P*-value^b^ by multivariable negative binomial regression adjusted for age, gender, creatinine, and comorbidities. *P* < 0.05 is statistically significant. IRR, incidence rate ratio; 95% CI, 95% confidence interval; LoS, length of stay.

## Discussion

In this investigation, hArg was discovered to be a unique biomarker connected to the severity of sepsis, hospital, and ICU LoS. When combined with SOFA scores, hArg may help to enhance ED triage decisions and hospital resource allocation. We used deep phenotyping approach to characterize OS and multiplex mass spectrometry to analyze plasma from patients with suspected infection and sepsis who presented at the most proximal timepoint in the hospital (i.e., the ED) prior to any treatment being rendered. This strategy was applied to investigate both systemic metabolic disturbances and potential mechanisms that lead to vascular derangements. The plasma metabolic profile first identified metabolites from the arginine-NO pathway, specifically hArg, and subsequently from the urea cycle and amino acid metabolism as being involved in vascular endothelial dysfunction. This multiplex approach confirmed the role of NO metabolism and identified a potential novel metabolite that could be useful for future clinical prediction.

The main findings were that hArg and endogenous inhibitors methylarginines (ADMA, SDMA, and NMMA) were associated with sepsis severity and LoS independently. (1) In multivariate analysis, Plasma hArg and its ratio with methylarginines (hArg: ADMA, hArg: SDMA, and hArg: NMMA) strongly predict ICU admission and LoS in the patient cohort. (2) In comparison to non-sepsis infection, plasma hArg: SDMA was significantly lower in sepsis patients and was strongly correlated with SOFA score and systemic inflammatory markers. (3) At the time of admission, plasma hArg: SDMA distinguished sepsis from infection. In comparison to Winkler et al. (16), we demonstrated that hArg and methylarginines improved the diagnostic and prognostic accuracy for sepsis when combined with SOFA score. Consistent with earlier research (19), we confirmed that hArg is useful for risk classification and that increased ADMA and SDMA are associated with poor outcomes.

Decreased arginine (Arg) elevated ADMA and SDMA levels, as well as low hArg: ADMA and hArg: SDMA plasma ratios induce cytotoxicity in endothelial cells (20). Excessive NO production can lead to septic shock (21–23). Sepsis can be difficult to distinguish from infection and non-infectious systemic inflammatory response syndrome (SIRS) because they exhibit similar signs and symptoms (24). We found that three plasma metabolites (glutamine, arginine, and hArg) potentially identified sepsis in control (Figure 2A) and infection (Figure 2B) patients and improved when combined with SOFA score (*P* < 0.001). Furthermore, hArg plasma levels were significantly lower in sepsis compared to controls and infection (Supplementary Figure 5B and Supplementary Table 5) and correlated positively with baseline diastolic blood pressure (DBP) (Supplementary Table 6), indicating that low circulating levels are associated with endothelial dysfunction.

Preclinical and prospective cohort studies (25, 26) have demonstrated the predictive relevance (27–32), and therapeutic efficacy of homoarginine (hArg) in sepsis patients. Plasma ADMA and SDMA levels have been linked to the severity of sepsis, organ dysfunction, and mortality in ICU patients (33, 34). Moreover, increased dimethylarginine catabolism and endogenous suppression of NOS are independent of glucose metabolism (35). We showed that plasma hArg, ADMA, and SDMA are prognostic markers of sepsis, and that hArg and hArg: SDMA ratios were two times lower in ICU patients than in hospital ward patients (*P* = 0.008, Supplementary Table 1). In accordance with previous studies, high plasma ADMA and SDMA levels are detrimental to vascular endothelial dysfunction, and SDMA, but not ADMA, is a specific, independent predictor of sepsis severity identifies patients at risk.

In addition, a low (25th) percentile baseline plasma hArg and methylarginines ratios were independently associated with ICU admission (Table 2) and LoS (Table 3). Among the three methylarginines (ADMA, SDMA, and NMMA), ADMA was the best predictor of ICU admission and LoS, and patients with low (25th) percentile plasma hArg:ADMA having two-fold increased likelihood of ICU admission and 2.62-fold LoS.

A high turnover of Arg and hArg induces OS and bioenergetic failure (36). Arginase regulates the urea cycle, and increased plasma arginase (ARG) is linked to low arginine bioavailability (37). hArg is an inhibitor of arginase (38, 39). We measured arginase to ornithine urea ratio (AOR) and showed a significant correlation between plasma hArg and AOR (adjusted partial correlation, *r* = 0.355, *P* < 0.001) (Supplementary Figure 5K and Supplementary Table 5). Furthermore, consistent with a previous kinetic study (38) a significant correlation was also found between lysine (endogenous ARG inhibitor) and AOR (partial correlation, *r* = 0.411, *P* < 0.001) (Supplementary Figure 5L and Supplementary Table 5) in sepsis group. Overall, we have demonstrated that decreased hArg and dimethylarginine levels, as well as their ratio, are predictors of sepsis outcomes (16, 19), and warrant further investigation.

Strengths of our study were (1) we implemented stringent quality assurance and QC measures in pre-processing and targeted measurement of metabolomics analytes to minimize variability. (2) The study was conducted in a well-defined cohort of patients with infection and sepsis representative of patients at the ED prior to any treatment rendered. The predictive model was developed with all patients surviving to the end of the study, thus minimizing dropout effects which would bias our LoS calculation, short-term outcome prediction, and diagnostic performance estimates.

Our study limitations were (1) the study was performed in a single site and recruited relatively small numbers of patients. Nonetheless, we minimized the potential bias results with proper selection protocol, data collection, and quality control for measurements and evaluation. (2) The study is a prospective, observational design which evaluated the association of biomarkers with patient outcome, not causality. (3) The study did not follow up patients for long-term sepsis symptoms and survival after hospital discharge. (4) Sepsis patients were predominantly male, which may skew our statistical interpretation (40). Lastly, there was only a small window of opportunity for recruitment of patients in the fast-paced ED with short turnaround time, since the local IRB mandated informed consent to be obtained from all subjects prior to sampling. Thus, almost all recruited subjects had mental capacity to provide the informed consent, which could have introduced spectrum bias of patients less likely to succumb from infection and sepsis. Nevertheless, we believe our data remains relevant to undifferentiated patients wherein diagnosis of sepsis and its severity are not clinically apparent at the onset.

## Conclusion

In conclusion, we examined the plasma metabolic profiles of patients with suspected infection and sepsis at the ED. We showed that hArg may be useful to diagnose and predict septic outcomes. The findings contribute to a better understanding of the role of hArg and NO metabolism in sepsis.

## Data availability statement

The original contributions presented in this study are included in the article/Supplementary material, further inquiries can be directed to the corresponding author.

## Ethics statement

The studies involving human participants were reviewed and approved by the NUHS Institutional Review Board. The patients/participants provided their written informed consent to participate in this study.

## Author contributions

WK and the clinic staff initiated the study, subjects’ recruitment, data collection, and followed up patients. MN performed the biostatistical analysis, interpreted the data, and wrote the manuscript. LP performed the metabolic profiling of patient samples. LW and EG performed the data processing and quality control. WK designed the clinical study and together with CD supervised the study and data analysis. All authors read and approved the final manuscript.
