# Supplementary material for: Deep phenotyping of oxidative stress in emergency room patients reveals homoarginine as a novel predictor of sepsis severity, length of hospital stay, and length of intensive care unit stay
Source: Front Med (Lausanne). 2022 Nov 25;9:1033083. doi: 10.3389/fmed.2022.1033083 (PMC9733670; doi:10.3389/fmed.2022.1033083)
Supplement: Supplementary file 1 [file Data_Sheet_1.PDF]

Supplementary Material

1 Supplementary Data

Chemicals and reagents for LC/MS-MS

Solvents of LC-MS grade such as Acetonitrile, Deionized water were purchased from Aik Moh Chemicals Pte. Ltd. (Singapore). Ergothioneine (ET; purity > 98%), Hercynine, S-Methylergothionine, and stable isotopes of Ergothioneine and Hercynine were purchased from Tetrahedron (Paris, France; www.tetrahedron.fr). Unlabelled amino acids/bile acid mix, and stable isotopic amino acids/bile acids mix were purchased from Cambridge Isotopes laboratories (Massachusetts, USA). Formic acid and Ammonium Formate, and most unlabelled compounds including Homoarginine, Nitrosoglutathione, 3-Nitrotyrosine, and Nitroarginine were purchased from Sigma-Merck (Singapore). Stable-isotope compounds such as Allantoin, Asymmetrical dimethyl arginine (ADMA), Butyrl-L-carnitine, Creatinine, Glutamine, Homocysteine, Homoarginine, Inosine, Isovaleryl-L-carnitine, Kynurenic acid, Vitamin B3, Propionyl-L-carnitine, Symmetrical dimethyl arginine (SDMA), Spermidine, Uric acid, and Xanthine were also purchased from Sigma-Merck (Singapore). 7α-hydroxy-4-Cholesten-3-one-d7, 8-iso Prostaglandin F2α, and 7α,12α-Dihydroxycholest-4-en-3-one-d7 were purchased from Cayman Chemical (Michigan, USA). Human plasma was purchased from i-DNA Biotechnology Pte. Ltd. (Singapore).

Sample Preparation for LC/MS-MS

This study used samples from clinical cohorts with matched controls. 50µL of plasma samples, calibrators, and quality control samples were transferred into a deep well 96-well plate, and then supplemented with 50µL of stable isotope internal standards mixtures at 5µg/L. All samples were

treated with 360µL of ice-cold Acetonitrile containing 0.1% Formic acid, mixed on a shaker at 1000 rpm/min for 10 minutes, and centrifuged at 2270 x g for 50 minutes at 4°C. 140µL of the supernatant was transferred onto a 96-well plate and loaded into the 7°C autosampler for LCMS/MS analysis. The MRM chromatogram peak area was then normalized against the stable isotope standard before being quantified using the standard curve.

LC/MS-MS Analysis

Multiple reaction monitoring (MRM) methods, analyses, and data acquisition using Agilent's 6495 tandem triple quadrupole mass spectrometer equipped with electrospray ionization (ESI) source in MRM mode and HILIC chromatographic conditions were as follows: Liquid chromatographic (LC) separation of the biomarkers was carried out on an Agilent 1290 Infinity II LC system with PEEK coated SeQuant®ZIC®-cHILIC 3mm, 100Å 100 x 2.1 mm HPLC column (Merck Pte Ltd, Singapore) maintained at 40°C. The organic solvent was Acetonitrile containing 0.1% Formic acid (Solvent A) and the aqueous solvent used was 20 mM Ammonium Formate pH 4.0 (Solvent B). A linear LC gradient on Binary Pump (Agilent Model G7120A) was set up with the percentage of Solvent B as follows: 10% at 0 minutes, 70% at 9.00 minutes, 70% at 11.0 minutes, and 10% between 11.1 and 11.5minutes, with a flow rate of 0.4 mL/min. The column was further equilibrated for further 11.5 minutes with 10% Solvent B. An additional high-speed pump, Binary Pump B (Agilent Model G7120A), together with a Quick-Change valve head, 2-position/10-port, 1,300 bar (Agilent Part No: 5067-4240), were utilized to reduce the cycle times by automated alternating column regeneration. % of Solvent B on Binary Pump B was maintained at 10% with a flow rate of 0.4 mL/min. The sample injection volume was 10µL. For mass detection, the LC eluent was connected online to an Agilent 6495 Triple Quadrupole MS system (Instrument model G6495A, Agilent Technologies) operated with the electrospray source in either positive or negative ionization mode. The electrospray ionization

source conditions were as follows: capillary voltage of 4.0 kV, nozzle voltage of 500 V, iFunnel parameter high/low-pressure RF of 90V, nebulizer pressure of 60 psi, the gas temperature of 290°C, sheath gas temperature of 350°C, Nebulizer was 35 psi, and sheath gas flow of 12L/min. Acquisitions and quantitative analysis were performed using the Agilent MassHunter Workstation Acquisition (10.0.127) and Agilent MassHunter Workstation Quantitative Analysis (10.0) software. Analytical workflow (Phase 0) was shown in the **Supplemental Fig.1**.

OS Assay Performance

We applied a multiplex mass spectrometry panel (**Supplemental Table 2**) followed by a multi-step quality control and analysis plan to identify key markers of OS that predict severity of sepsis and clinical outcomes, which resulted in 269 qualified chromatographic features. A total of 62 batch quality control (BQC) samples were pooled from 300 individual samples. Dispersion ratio (D-ratio ≤ 50%) and coefficient of variance (CoV ≤ 30%) were calculated from repeat measurement of pooled BQC and used as acceptance criteria (Phase 1a) in data pre-processing (**Supplemental Fig. 1**).

Mass Spectrometry Panel, Quality Control and Quality Assessment

A semi-targeted, multiplex panel for the assessment of OS was assembled from a combination of literature references and known biochemical pathways. Metabolites are categorized by class of OS marker in addition to their signalling pathway. The raw chromatographic data acquired from Agilent MassHunter were then converted into the standard mzML 1.1.0 format (1). ProteoWizard msConvert was used to perform this conversion (2). The MRMKit was used for automated background subtraction, area peak picking, retention time alignment, peak integration, inter-batch correction, and intra-batch wavelet transforms smoothening of integrated peak intensity, and analyte:IS response ratio computation (3). Data processing (Phase 1) were detailed in the **Supplementary Fig.1**.

2. Supplemental Results

Pairwise Comparison Ranking and Dimensionality Reduction of the Measured Data

Using an initial criterion of ±1 fold change (FC) and a Bonferroni adjusted p-value of < 0.05 (independent sample t-test), 114 metabolites were identified as differences between sepsis and control groups and 58 analytes were identified as differences between sepsis and infection groups (**Supplemental Table 3, Supplemental Table 4**). Repeating the analysis with increased stringency (±2 FC, adjusted p-value < 0.0001), 20 and 22 metabolites met the stringent criteria for sepsis vs control and sepsis vs infection comparisons, respectively (**Fig. 3A, Fig. 3B, Supplemental Table 5**). In these metabolites, we found that NO related metabolites generally had the highest statistical significance and accounted for 6 of 10 in the sepsis vs. control and 3 of 10 in the sepsis vs. infection comparisons. In the full metabolite panel list (**Supplemental Table 2**), we analysed 19 of 269 unique metabolites from the nitric oxide (NO) pathway. Matrix based analyses were first performed using principal component analysis (PCA). PCA did not reveal any outliers outside the 95% CI of the Hotelling's T-squared distribution. The PCA score plot represents a weighted average of all variables (metabolites) of the first two principal components (PC-1, PC-2) responsible for separation between the two groups (sepsis vs control), finding that 13.70% (PC-1) and 12.30% (PC-2) (26% cumulatively) of variance between sepsis and control patients were accounted for by the first two principal components (PCs), respectively (**Supplemental Fig. 3A**). Likewise, 15% (PC-1) and 9.10% (PC-2) (24.10% cumulatively) were accounted for by the first two PCs when comparing patients with sepsis versus those with infection (**Supplemental Fig. 3B**). In consideration of the ratio of biomarker variables (~104) to the number of observations (~102) within a predictor matrix, we modelled the covariance between matrices using a supervised partial least squares discriminant analysis (PLS-DA) based on the optimal components determined by separation distance, i.e., the ratio of the between group sum of the squares and the within group sum of squares (B/W-ratio). The projections of maximum variance within the hyperplane were

then ranked by each component score (composed of a weighted sum of squares of the PLS loadings for Y-variation in each dimension). Group discrimination along the axes of three principal components (PLS-1, PLS-2) in the score plot, finding 13.10% and 4.50% for sepsis vs control (**Supplemental Fig. 3E**) and 10.40% and 11.60% for sepsis vs infection (**Supplemental Fig. 3F**). Features with a variable importance of projection (VIP) score of > 2 were selected as candidate markers for sepsis discrimination (**Supplemental Fig. 3G, Supplemental Fig. 3H).** Cross-correlation matrices of the top 10 VIP features discriminating between the two groups were then visualised by hierarchical clustering heatmap (**Supplemental Fig. 3I, Supplemental Fig. 3J**). the heatmaps show a lower intensity of NO markers in sepsis, namely lysine, glutamine, hArg, Arg, citrulline, and acetylornithine, whereas higher intensity of endogenous inhibitors of NO synthesis (SDMA, ADMA, NMMA). These key features were further selected using sparse PLS-DA (sPLS-DA) (4) and yielded 4 NO pathway metabolites (5 ranked): lysine, glutamine, hArg, and Arg (sepsis vs control) (**Supplemental Fig. 3K**) and SDMA (sepsis vs infection) (**Supplemental Fig. 3L**). The PLS-DA model was validated using a 10-fold cross validation (CV) error rate calculation on the selected features component. The model showed goodness-of-fit (Q2 = 0.62, R2Y = 0.79, difference between R2Y and Q2 <0.3) with an accuracy of 0.93, (sepsis vs control) and (Q2 = 0.27, R2 = 0.45) with accuracy of 0.78 (sepsis vs. infection). The model overfitting was tested by permutation testing with an empirical p-value < 5e-04 (0/2000 test).

Segmented Analyses Within NO Pathway Markers

In the segmental analysis we focused on the metabolites NO metabolites panel. The univariate and multivariate analyses revealed 5 metabolites (lysine, glutamine, hArg, Arg, and SDMA) as robust markers differentiated between the non-infectious control, infection and sepsis (**Supplemental Fig. 4A, Supplemental Fig. 4B, Supplemental Fig. 4C, Supplemental Fig. 4D**). [Goodness-of-fit (Q2 = 0.51, R2Y = 0.53, difference between R2Y and Q2 < 0.3), accuracy = 0.89]. The ROC-AUC for PLS-

DA model showed good discrimination for sepsis vs control (ROC-AUC = 0.99, P < 0.001) and sepsis vs infection (ROC-AUC = 0.83) **(Supplemental Fig. 4E, Supplemental Fig. 4F**).

References

1. Martens L, Chambers M, Sturm M, Kessner D, Levander F, Shofstahl J, et al. mzML--a community standard for mass spectrometry data. Mol Cell Proteomics. 2011;10(1):R110.000133.

2. Chambers MC, Maclean B, Burke R, Amodei D, Ruderman DL, Neumann S, et al. A cross-platform toolkit for mass spectrometry and proteomics. Nat Biotechnol. 2012;30(10):918-20.

3. Teo G, Chew WS, Burla BJ, Herr D, Tai ES, Wenk MR, et al. MRMkit: Automated Data Processing for Large-Scale Targeted Metabolomics Analysis. Anal Chem. 2020;92(20):13677-82.

4. KA LC, Boitard S, Besse P. Sparse PLS discriminant analysis: biologically relevant feature selection and graphical displays for multiclass problems. BMC Bioinformatics. 2011;12:253.

Supplemental Table 1. SOFA components, clinical characteristics, and significant metabolites in hospital and ICU patients assessed at ED and 24hrs post admission

Variables	Hospital (N = 290)	ICU (N = 24)	P-value [a]
Presence of acute organ failure, n (%)	6 (2.33)	9 (37.50)	<0.001
**LoS**			
hospital	5.37±5.23	15.96±13	<0.001
ICU	0.03±0.44	6.21±5.81	<0.001
**SOFA components**			
**Coagulation**			
Platelet count (x109/L) (ED)	240.82±91.75	256.87±133.23	0.422
Platelet count (x109/L) (24hr)	239.46±116.63	234.82±136.85	0.861
**Renal**			
Creatinine, (µmol/L) (ED)	111.43±141.58	187.25±213.01	0.015
Creatinine >176 µmol/L (ED), n (%)	26 (10.16)	5 (20.83)	0.019
Creatinine, (µmol/L) (24hr)	101.04±108.40	172.86±211.24	0.013
Creatinine >176 µmol/L (24hr), n (%)	12 (4.66)	4 (17.39)	0.074
**Liver**			
Bilirubin (µmol/L) (ED)	14.90±12.97	13.68±7.61	0.713
Bilirubin >34 µmol/L (ED), n (%)	11 (4.28)	0	0.309
Bilirubin (µmol/L) (24hr)	16.26±19.25	12.30±10.93	0.471
Bilirubin >34 µmol/L (24hr), n (%)	7 (2.72)	1 (7.69)	0.974
**Respiratory**			
Oxygen saturation (%)	97.31±3.64	95.58±3.80	0.025
Fractional inspired oxygen (%)	21.79±3.69	28.45±12.72	<0.001
PaO2/FiO2 or SpO2/FiO2 ratio	356.31±126.31	228.88±100.51	0.001
P/F ratio <300	0.21±0.41	0.71±0.46	<0.001
Intubation, n (%)	0	5 (20.80)	<0.001
**Cardiovascular**			
Vasopressor treatment, n (%)	1 (0.39)	4 (16.66)	<0.001
Mean arterial pressure (MAP) (mmHg) (ED)	93.82±14.10	82.55±15.28	<0.001
MAP ≤ 70mmHg, n (%)	11 (4.28)	5 (20.80)	<0.001
MAP > 70mmHg, n (%)	250 (97.27)	19 (79.20)	
Systolic blood pressure (ED)	130.27±23.25	116±24.14	0.004
Diastolic blood pressure (ED)	75.59+11.73	65.83+12.63	<0.001
Widened blood pressure (PP)	54.68±18.12	50.16±17.36	0.236
Heart rate (ED)	105.50±16.94	115.25±25.41	0.009
Diastolic shock index (DSI)	1.42±0.29	1.80±0.50	<0.001
**CNS**			
Altered mental state, n (%)	257 (100)	24 (100)	1
**Clinical characteristics**			
INR (mmol/L)	55.03±13.95	54.79±14.31	0.936
Blood glucose (mmol/L)	1.40±0.49	1.27±0.14	0.298
HbA1c(mmol/L)	9.66±5.24	10.44±5.09	0.495
IPTT (sec) (ED)	8.23±2.72	7.65±2.11	0.468
IPTT (sec) (24hr)	39.76±13.16	37.67±7.23	0.551
**Clinical risk scores**			
CMI	1.80±1.78	2.25±2.73	0.256

MEDS score	2.19±2.45	4.17±3.42	<0.001
ED SOFA score	1.10±1.46	2.92±1.81	<0.001
Highest SOFA score in-hospital stay	0.77±1.34	3.92±3.17	<0.001
Worst SOFA score overall	1.28±1.60	4.25±3.01	<0.001
Day of occurrence of worst SOFA score overall (days)	0.28±1.34	4.04±8.01	<0.001
Quick SOFA score (qSOFA)	0.34±0.51	0.58±0.71	0.034
qSOFA<2	252 (98.05)	21 (87.50)	
qSOFA>2	5 (1.94)	3 (12.50)	
Deteriorate SOFA from ED, n (%)	28 (10.89)	13 (54.20)	<0.001
**Site of infection, n (%)**			0.004
Respiratory	76 (29.57)	10 (41.70)	
Urinary	51 (19.84)	1 (4.20)	
Gastrointestinal	20 (7.78)	0	
Hepatobiliary	15 (5.83)	6 (25)	
Skin	47 (18.28)	3 (12.50)	
Others	41 (15.95)	4 (16.70)	
Unknown	17 (6.61)	0	
Treatment, n (%)			
Oral antibiotics	128 (49.80)	9 (37.50)	0.343
Intravenous antibiotics	227 (88.32)	24 (100)	0.036
Antibiotic escalation	18 (7)	5 (20.80)	0.043
Vasopressor	1 (0.39)	4 (16.70)	<0.001
Amount of intravenous fluid	1162.81±929.68	1698.14±1242.48	0.012
**Significant metabolites**			
Lysine	13.59±4.30	12.11±4	0.103
Glutamine	0.11±0.08	0.23±0.23	<0.001
Arg	282.06±130.02	287.47±174.47	0.847
hArg	10.09±7.45	6.01±4.53	0.008
hArg:SDMA	1040.16±906.55	512.94±504.54	0.005

Continuous data are means±SD, and categorical data are frequency (percentage), n (%). Percentile value was log-transformed.

P-value [a] was determined by independent Student's t-test for numerical variables, or χ^2 test for categorical variables. $P < 0.05$ is statistically significant. Abbreviations: SOFA score sequential organ failure assessment score, MAP mean arterial pressure, CNS central nervous system, PaO2/FIO2 ratio of arterial oxygen partial pressure, PaO2 (mmHg) to fractional inspired oxygen, SaO2/FIO2 ratio of SaO2 peripheral arterial oxygen saturation to fractional inspired oxygen. Altered mental state was assessed by GlasgowComa Scale (GCS) for neurological dysfunction. hArg homoarginine; SDMA symmetric dimethylarginine, and hArg:SDMA plasma hArg/SDMA ratio.

Supplemental Table 2. SRM acquisition for all metabolites and internal standards

PUBCHEM ID	Metabolites	MW (g/mol)	Precursor Ion (*m/z*)	Product Ion (m/z)	Ret. Time (min)	Collision Energy (V)	Cell Acc. Voltage (V)	Polarity	KEGG ID	Compound class / Major Pathway
6274	Histidine	155.2	156.1	110.1	6.04	14	5	Pos	C00135	Amino acids
199	Agmatine	130.2	131	72	3.29	15	5	Pos	C00179	Amino acids
6262	Ornithine	132.2	133.1	70.1	5.1	16	5	Pos	C00077	Amino acids
145742	Proline	115.1	116.1	70.1	3.45	13	5	Pos	C00148	Amino acids
5962	Lysine	146.2	147	84	6.1	14	5	Pos	C00047	Amino acids
597	Cytosine	111.1	112	95	6.1	20	5	Pos	C00380	Amino acids
750	Glycine	75.1	76.4	58.1	3	10	5	Pos	C00037	Amino acids
5950	Alanine	89.1	90.1	44	4.22	6	5	Pos	C00041	Amino acids
5951	Serine	105.1	106	60	2.49	15	3	Pos	C00065	Amino acids
6106	Leucine	131.2	132.1	86	2.83	8	5	Pos	C00123	Amino acids
6140	Phenylalanine	165.2	166	120	2.71	10	5	Pos	C00079	Amino acids
6287	Valine	117.2	118	72	3.43	8	5	Pos	C00183	Amino acids
6288	Threonine	119.1	120	74.1	4.34	10	5	Pos	C00188	Amino acids
6306	Isoleucine	131.2	132.1	86	2.83	8	5	Pos	C00407	Amino acids
99288	Alloisoleucine	131.2	132.1	43.1	2.08	20	5	Pos	n.a	Amino acids
46878393	Orotidine-5-phosphate	365.2	367	323	0.74	18	5	Neg	n.a	Amino acids
970	Oxaloacetate	132.1	131	87	1.57	8	5	Neg	C02362	Asparagine Metabolism
164533	2-Ketoglutarate	146.1	145	101	2.1	13	5	Neg	n.a	Asparagine Metabolism
5960	Aspartate	133.1	134.3	74	3.4	12	5	Pos	C00049	Asparagine Metabolism
5961	Glutamine	146.1	147.1	84.1	6.1	17	5	Pos	C00064	Asparagine Metabolism
33032	Glutamic acid	147.1	148.2	84.2	4.7	15	5	Pos	C00025	Asparagine Metabolism
6267	Asparagine	132.1	133.1	74	4.4	13	5	Pos	C00152	Asparagine Metabolism
9903	Lithocholic acid (LCA)	376.6	375.5	375.5	1.01	20	5	Neg	n.a	Bile acids
10140	Glycocholic acid (GCA)	465.6	464.4	74.2	1.67	30	5	Neg	C01921	Bile acids
12544	Glycochenodeoxycholic acid (GCDCA)	449.6	448.3	74	1.6	10	5	Neg	n.a	Bile acids
115245	Glycolithocholic acid (GLCA)	433.6	432.3	73.9	0.94	10	5	Neg	n.a	Bile acids
123743	7?-Hydroxy-4-cholesten-3-one (7-HCO)	400.6	401.5	177	0.65	24	5	Pos	n.a	Bile acids

221493	Cholic acid (CA)	408.6	407.2	345.2	0.69	32	5	Neg	C00695	Bile acids
222528	Deoxycholic acid (DCA)	392.6	391.2	345.2	0.82	36	5	Neg	C02528	Bile acids
387316	Taurochenodeoxycholic Acid (TCDCA)	499.7	498.3	498.3	0.7	20	5	Neg	n.a	Bile acids
439763	Taurolithocholic acid (TLCA)	483.7	498.3	80	2.12	70	5	Neg	n.a	Bile acids
2733768	Taurodeoxycholic Acid (TDCA)	499.7	498.3	498.3	2	20	5	Neg	n.a	Bile acids
3035026	Glycodeoxycholic acid (GDCA)	449.6	448.3	74	1.6	10	5	Neg	n.a	Bile acids
3081958	7?,12?-Dihydroxycholest-4-en-3-one (7,12-DHCO)	416.6	417.1	381.2	0.86	20	5	Pos	n.a	Bile acids
5283820	Hyodeoxycholic acid (HDCA)	392.6	391.3	391.3	5.8	30	5	Neg	n.a	Bile acids
12310288	Glycoursodeoxycholic acid (GUDCA)	449.6	448.3	74	1.6	10	5	Neg	n.a	Bile acids
288	Free carnitine (C0)	161.2	162	103	3.54	22	5	Pos	n.a	Carnitines/ Acyl carnitines
461	Hexadecanoylcarnitine (C16)	399.6	400	85.1	1.02	45	5	Pos	C02990	Carnitines/ Acyl carnitines
725	Deoxycarnitine	145.2	146.2	87	3.3	16	5	Pos	n.a	Carnitines/ Acyl carnitines
123701	Octanoylcarnitine (C8)	287.4	288	85.1	1.4	30	5	Pos	n.a	Carnitines/ Acyl carnitines
168381	Dodecanoylcarnitine (C12)	343.5	344	85.1	1.02	39	5	Pos	n.a	Carnitines/ Acyl carnitines
188824	Propionylcarnitine (C3)	217.3	218.1	85	2.3	15	5	Pos	n.a	Carnitines/ Acyl carnitines
439829	Butyrylcarnitine (C4)	231.3	232	85.1	1.84	20	5	Pos	n.a	Carnitines/ Acyl carnitines
6426851	Isovaleryl-carnitine (3-M-C4:0)	245.3	246.1	85.1	1.52	15	5	Pos	n.a	Carnitines/ Acyl carnitines
6426853	Hexanoylcarnitine (C6)	259.3	260	85.1	1.61	29	5	Pos	n.a	Carnitines/ Acyl carnitines
6441392	Octadecenoylcarnitine (C18:1)	425.6	426	85.1	1.03	40	5	Pos	n.a	Carnitines/ Acyl carnitines
6450015	Decadienylcarnitine (C10:2)	423.6	312	85.1	1.4	35	5	Pos	n.a	Carnitines/ Acyl carnitines
7045767	Acetylcarnitine (C2)	203.2	204.3	85.1	1.6	26	5	Pos	n.a	Carnitines/ Acyl carnitines
10094444	Behenic carnitine (C22:0)	485	484.7	85.1	0.74	25	5	Pos	n.a	Carnitines/ Acyl carnitines
10245190	Decanoylcarnitine (C10)	315.4	316	85.1	1.3	36	5	Pos	n.a	Carnitines/ Acyl carnitines
16226475	Valeryl-carnitine (C5)	245.3	246	85.1	1.56	28	5	Pos	C20826	Carnitines/ Acyl carnitines
22833575	Cis-5-tietradecenoylcarnitine	369.5	370.3	85	0.58	15	5	Pos	n.a	Carnitines/ Acyl carnitines
28480372	Arachidic carnitine (C20:0)	457	456.7	85.1	0.93	25	5	Pos	n.a	Carnitines/ Acyl carnitines
50896251	Succinylcarnitine (C4-DC)	262.3	262	85.1	2.7	22	5	Pos	n.a	Carnitines/ Acyl carnitines
52922056	Octadecanoylcarnitine (C18)	427.7	428	85.1	1.03	47	5	Pos	n.a	Carnitines/ Acyl carnitines
53477791	Myristoylcarnitine (C14:0)	371.6	372.3	85.1	1.62	30	5	Pos	n.a	Carnitines/ Acyl carnitines
71296139	Adipoylcarnitine (C6DC)	289.3	290	85.1	2.84	35	5	Pos	n.a	Carnitines/ Acyl carnitines

71317118	Glutarylcarnitine (C5DC)	275.3	276	85.1	3.22	40	5	Pos	n.a	Carnitines/ Acyl carnitines
71317118	Glutarylcarnitine (C6-OH)	275.3	276	85.1	3.6	40	5	Pos	n.a	Carnitines/ Acyl carnitines
71464474	3-hydroxyisovalerylcarnitine (C5-OH)	261.3	262	85.1	2.69	35	5	Pos	n.a	Carnitines/ Acyl carnitines
71464477	Hydroxybutyryl-carnitine (C4-OH)	247.3	248	85.1	2.98	30	5	Pos	n.a	Carnitines/ Acyl carnitines
71464541	3-Hydroxyltetradecanoyl	387.6	388	85.1	1.6	40	5	Pos	n.a	Carnitines/ Acyl carnitines
71464579	Hydroxydodecenoylcarnitine	357.5	358.5	85.1	1.16	25	5	Pos	n.a	Carnitines/ Acyl carnitines
71752411	Tetradecenoylcarnitine (C14:1)	369.5	370.3	85.1	1.23	23	5	Pos	n.a	Carnitines/ Acyl carnitines
86583357	3-Hydroxyoctanoylcarnitine (C8-OH)	303.4	304	85.1	2	35	5	Pos	n.a	Carnitines/ Acyl carnitines
91825606	Malonylcarnitine (C3-DC)	247.2	248.2	85.1	3.08	32	5	Pos	n.a	Carnitines/ Acyl carnitines
129628702	Decenoylcarnitine (C10:1)	313.4	314	85.1	1.3	35	5	Pos	n.a	Carnitines/ Acyl carnitines
129664620	Dodecenoylcarnitine (C12:1)	341.5	342.5	85.1	1.16	25	5	Pos	n.a	Carnitines/ Acyl carnitines
129673643	Methylcrotonylcarnitine (C5:1)	243.3	244	85.1	1.66	30	5	Pos	n.a	Carnitines/ Acyl carnitines
129691756	Tetradecadienoylcarnitine (C14:2)	367.5	368.3	85.1	1.42	28	5	Pos	n.a	Carnitines/ Acyl carnitines
129692230	Octenoylcarnitine (C8:1)	285.4	286	85.1	1.43	24	5	Pos	n.a	Carnitines/ Acyl carnitines
129846791	Hexenoylcarnitine (C6:1)	257.3	258	85.1	1.6	35	5	Pos	n.a	Carnitines/ Acyl carnitines
129892586	Hydroxyhexadecenoylcarnitine (C16:1-OH)	413.6	414	85.1	1.02	45	5	Pos	n.a	Carnitines/ Acyl carnitines
132277610	Octadecadienylcarnitine (C18:2)	409.6	410.1	85.1	1.02	40	5	Pos	n.a	Carnitines/ Acyl carnitines
247	Betaine	117.2	118	58	3.02	25	5	Pos	n.a	Choline metabolism
305	Choline	104.2	104	60	2.36	17	5	Pos	C00114	Choline metabolism
673	Dimethylglycine	103.1	104.1	58.1	2.49	10	5	Pos	C01026	Choline metabolism
657272	Glycerophosphocholine	257.2	258.1	104	4.44	16	5	Pos	C00670	Choline metabolism
586	Creatine	131.1	132.1	44.3	2.94	21	5	Pos	C00300	Creatine Metabolism
588	Creatinine	113.1	114	44.3	2.23	21	5	Pos	C00791	Creatine Metabolism
217324	Guanidineacetate	117.1	118	76	4.37	10	5	Pos	n.a	Creatine Metabolism
9548602	Phosphocreatine	211.1	212	90.1	5	14	5	Pos	C02305	Creatine Metabolism
1145	Trimethylamine oxide (TMAO)	75.1	76	58	3.17	25	5	Pos	n.a	Dietary Metabolites
1146	Trimethylamine (TMA)	59.1	60.1	44.1	2.94	30	6	Pos	n.a	Dietary Metabolites
5351619	Ergothioneine	229.3	230	127	3.22	20	5	Pos	n.a	Dietary Metabolites
5459798	Hercynine	197.2	198	154.1	5	9	4	Pos	C05575	Dietary Metabolites
10105842	S-methyl ergothioneine	243.3	244.1	141	3.52	20	5	Pos	n.a	Dietary Metabolites

681	Dopamine	153.2	154.2	91.2	1.15	22	5	Pos	n.a	Endocrine function
1237	Normetanephrine	183.2	184.2	134.1	1.38	20	5	Pos	n.a	Endocrine function
1669	3-Methoxytyramine	167.2	168	151	0.78	20	5	Pos	n.a	Endocrine function
5816	Epinephrine	183.2	184.1	166	1.8	11	5	Pos	C00788	Endocrine function
21100	Metanephrine	197.2	198.1	180.5	4.64	13	5	Pos	C05588	Endocrine function
439260	Noradrenaline	169.2	170.2	93	5.43	26	5	Pos	C00547	Endocrine function
5282263	8 Isoprostane	354.5	353.4	193.2	0.84	30	5	Neg	n.a	Lipid peroxidation metabolites
10415542	LPC 22:6	567.7	568.3	184	1.04	25	5	Pos	n.a	Lipid peroxidation metabolites
11757087	Lysophosphatidylcholine LPC 20:1	541.7	542.3	184	1.13	25	5	Pos	n.a	Lipid peroxidation metabolites
24779473	LPC 20:0	551.7	552.4	184	1.04	25	5	Pos	n.a	Lipid peroxidation metabolites
24779481	LPC 24:0	607.8	608.5	184	1.01	25	5	Pos	n.a	Lipid peroxidation metabolites
52924051	LPC 20:1	549.7	550.4	184	1.04	25	5	Pos	n.a	Lipid peroxidation metabolites
52924055	LPC 20:3	545.7	546.4	184	1.04	25	5	Pos	n.a	Lipid peroxidation metabolites
52924059	LPC 22:2	575.8	576.4	184	1.04	25	5	Pos	n.a	Lipid peroxidation metabolites
53480469	LPC 20:4	543.7	544.3	184	1.04	25	5	Pos	n.a	Lipid peroxidation metabolites
131750810	LPC 26:0	635.9	636.5	184	1.01	25	5	Pos	n.a	Lipid peroxidation metabolites
131750816	LPC 28:1	661.9	662.5	184	1.01	25	5	Pos	n.a	Lipid peroxidation metabolites
107904	Dityrosine	360.4	361.3	315.1	0.72	25	5	Pos	n.a	Modified Amino acids
110992	3-Chlorotyrosine	215.6	216.1	170	3.23	12	5	Pos	n.a	Modified Amino acids
89	3-Hydroxykynurenine	224.2	225.1	208.1	4.6	13	5	Pos	C02794	Modified Amino acids
144	5-Hydroxytryptophan	220.2	177.1	160.1	3.06	14	5	Pos	n.a	Modified Amino acids
779	Homoserine	119.1	120.2	44.2	3	32	5	Pos	n.a	Modified Amino acids
1088	Sarcosine	89.1	90.04	44.1	4.29	20	5	Pos	C00213	Modified Amino acids
5810	Hydroxyproline	131.1	132	85.9	2.95	14	5	Pos	C01015	Modified Amino acids
7405	5-Oxo-l-proline	129.1	128	84	3.12	5	5	Neg	n.a	Modified Amino acids
10972	N-acetylglycine	117.1	118.2	72.1	3.41	16	5	Pos	n.a	Modified Amino acids
12035	N-acetylcysteine	163.2	164	43.2	3.51	17	5	Pos	C06809	Modified Amino acids
21236	Norleucine	131.2	132.2	69.1	2.82	16	5	Pos	C01933	Modified Amino acids

25561	N-acetylglutamine	188.2	189.1	130	5.37	17	5	Pos	n.a	Modified Amino acids
36143	4-Hydroxyphenylglycine	167.2	168.1	123.1	0.79	18	5	Pos	n.a	Modified Amino acids
64969	3-Methylhistidine	169.2	170.1	126	5.89	12	5	Pos	n.a	Modified Amino acids
65040	5-Methylcytosine	125.1	126.2	109	4.6	20	5	Pos	C02376	Modified Amino acids
65065	N-acetylaspartate	175.1	176	134	4.18	10	5	Pos	C01042	Modified Amino acids
65065	N-acetylaspartic acid	175.1	174	88	3.39	12	5	Neg	C01042	Modified Amino acids
65098	Norvaline	117.2	118.2	72.1	3.16	16	5	Pos	C01799	Modified Amino acids
65249	N-acetylserine	147.1	146	116	3.4	4	5	Neg	n.a	Modified Amino acids
68144	Phenylacetylglycine	193.2	194.1	76	3.17	10	5	Pos	C05598	Modified Amino acids
68841	3-Phosphoserine	185.1	186	88	1.02	12	5	Pos	n.a	Modified Amino acids
69248	4-Hydroxyproline	131.1	132.1	86.1	2.97	10	5	Pos	C01015	Modified Amino acids
69520	3-Methylhistamine	125.2	126.2	96.1	5.87	21	5	Pos	n.a	Modified Amino acids
70914	N-acetylglutamic acid	189.2	190.2	130.1	5.4	12	5	Pos	n.a	Modified Amino acids
88064	N-Acetylalanine	131.1	130	88	1.92	14	5	Neg	C04341	Modified Amino acids
90638	Methylhistidine	169.2	170.1	96	5.89	22	5	Pos	n.a	Modified Amino acids
92105	1-Methylhistidine	169.2	170.1	124.1	5.54	14	5	Pos	n.a	Modified Amino acids
92832	N6-acetyl-L-lysine	188.2	189.1	126.1	5.77	10	5	Pos	n.a	Modified Amino acids
92859	3,5-Diiodo-L-thyronine	525.1	523.9	126.8	1.03	28	5	Neg	n.a	Modified Amino acids
97536	S-hexyl-glutathione	391.5	392.2	263	2.92	11	5	Pos	n.a	Modified Amino acids
99478	O-acetyl-L-serine	147.1	148.1	131.1	6.07	10	5	Pos	n.a	Modified Amino acids
123800	N(epsilon)-(carboxymethyl)lysine (CML)	204.2	205.1	84.1	4.7	25	5	Pos;	n.a	Modified Amino acids
439406	O-succinyl-L-homoserine	219.2	220.1	102.1	3.17	11	5	Pos	C01118	Modified Amino acids
440005	Nitroarginine	219.2	220.1	59.1	3.34	35	5	Pos	n.a	Modified Amino acids
1549098	3-Sulfino-L-alanine	153.2	154.1	122	2.2	10	5	Pos	n.a	Modified Amino acids
3032849	5-Hydroxylysine	162.2	163.2	145.2	3.41	10	5	Pos	n.a	Modified Amino acids
6992003	N6-Trimethyl-lysine	189.3	189.2	130.1	5.43	14	5	Pos	n.a	Modified Amino acids
10176265	N- acetylcysteine (NAC)	162.2	163.9	122	3.24	2	5	Pos	n.a	Modified Amino acids
22088197	N(epsilon)-(carboxyethyl)lysine	218.2	219	130.1	3.98	15	5	Pos	n.a	Modified Amino acids
53477713	Cysteineglutathione disulfide	426.5	427	298	0.48	15	5	Pos	n.a	Modified Amino acids
5886	NADP+	744.4	744.1	604.1	1.5	20	5	Pos	C00006	NAD+ metabolome

457	1-Methylnicotinamide	137.2	138.2	93.1	3.34	21	5	Pos	n.a	NAD+ metabolome
936	Nicotinamide	122.1	123	79.9	3.53	18	5	Pos	C00153	NAD+ metabolome
938	Nicotinic acid	123.1	124	80.1	5.37	18	5	Pos	C00253	NAD+ metabolome
14180	Nicotinamide ribotide	334.2	335	123	0.74	30	5	Pos	n.a	NAD+ metabolome
72924	6'-Hydroxynicotinate	139.1	140.1	94	3.51	22	5	Pos	n.a	NAD+ metabolome
123953	NAADP	745.4	745.1	604	1.01	20	5	Pos	C13051	NAD+ metabolome
249885	6-Hydroxynicotine	178.2	140.1	122.1	2.2	18	5	Pos	n.a	NAD+ metabolome
439924	Nicotinamide riboside	255.3	255.1	56.9	1.78	24	5	Pos	C03150	NAD+ metabolome
12243705	Methylnicotinamide	136.2	137	94	1.89	20	5	Pos	n.a	NAD+ metabolome
86587018	Nicotinamide oxide	275.3	139	79	3.27	20	5	Pos	n.a	NAD+ metabolome
6057	Tyrosine	181.2	182	136	3.74	8	5	Pos	C00082	NO related metabolites
65124	3-Nitrotyrosine	226.2	227.1	181.1	3.63	12	5	Neg	n.a	NO related metabolites
9085	Homoarginine (hArg)	188.2	189.2	144	5.89	15	5	Pos	n.a	NO related metabolites
9750	Citrulline	175.2	176.1	70.1	6.1	15	5	Pos	n.a	NO related metabolites
123831	Asymmetric dimethylarginine (ADMA)	202.3	203.15	46.2	4.88	17	5	Pos	C03626	NO related metabolites
132862	N-monomethylarginine (NMMA)	188.2	189.2	74	5.4	15	5	Pos	n.a	NO related metabolites
169148	Symmetrical dimethylarginine (SDMA)	202.3	203.15	171.9	4.8	9	5	Pos	n.a	NO related metabolites
6322	Arginine	174.2	175	70	6.11	25	5	Pos	C00062	NO related metabolites
649	5,6-Dihydrouracil	114.1	115.1	74.1	7.85	10	5	Pos	n.a	Nucleotides/ Modified Nucleotides
1044	Purine	120.1	121.1	94	2.71	21	5	Pos	n.a	Nucleotides/ Modified Nucleotides
1135	Thymine	126.1	127.1	110	6	19	5	Pos	n.a	Nucleotides/ Modified Nucleotides
1174	Uracil	112.1	113	70	2.2	20	5	Pos	C00106	Nucleotides/ Modified Nucleotides
6029	Uridine	244.2	245.1	113	2.14	8	5	Pos	C00299	Nucleotides/ Modified Nucleotides
6031	UDP	404.2	406.1	97.1	0.657	25	5	Pos	C00015	Nucleotides/ Modified Nucleotides
6175	Cytidine	243.2	244.1	112	3.2	14	5	Pos	n.a	Nucleotides/ Modified Nucleotides
13712	2-Deoxyuridine	228.3	229.1	113	3.4	10	5	Pos	n.a	Nucleotides/ Modified Nucleotides
27476	1-Methyladenosine	281.3	281.8	150	3.2	27	5	Pos	n.a	Nucleotides/ Modified Nucleotides

60961	Adenosine	267.2	268	136	3.1	17	5	Neg	n.a	Nucleotides/ Modified Nucleotides
64959	Xanthosine	284.2	285	153	0.6	10	5	Pos	C01762	Nucleotides/ Modified Nucleotides
65063	dUMP	308.2	307	195	0.85	18	5	Neg	C00365	Nucleotides/ Modified Nucleotides
65110	Aminoimidazole carboxamide ribonucleotide	338.2	339	110	2.2	32	5	Pos	n.a	Nucleotides/ Modified Nucleotides
193653	S-carboxymethyl-L-cysteine	179.2	180.1	163	2.9	10	5	Pos	n.a	Nucleotides/ Modified Nucleotides
439176	5-Methylthioadenosine	297.3	298.1	136.1	3.31	10	5	Pos	n.a	Nucleotides/ Modified Nucleotides
439176	S-methyl-5-thioadenosine	297.3	298	136	3.19	29	5	Pos	n.a	Nucleotides/ Modified Nucleotides
7059571	Cyclic-AMP	328.2	328	134.05	1.81	33	5	Neg	n.a	Nucleotides/ Modified Nucleotides
135398570	cyclic-GMP	345.2	346	152	0.82	20	5	Pos	n.a	Nucleotides/ Modified Nucleotides
135398593	Deoxyinosine	252.2	253.1	137	0.71	10	5	Pos	n.a	Nucleotides/ Modified Nucleotides
135398631	GMP	363.2	364	152	0.71	21	5	Pos	n.a	Nucleotides/ Modified Nucleotides
135398643	Inosine 5' triphosphate	508.1	508.9	344.9	8.3	10	5	Pos	C00081	Nucleotides/ Modified Nucleotides
135440064	8-Hydroxydeoxyguanosine	283.2	284.3	168.1	0.7	10	5	Pos	n.a	Nucleotides/ Modified Nucleotides
135445750	7-Methylguanosine	298.3	298	166	2.14	24	5	Pos	C20674	Nucleotides/ Modified Nucleotides
135767295	Oxoguanine	165.1	168	151	0.78	20	5	Pos	n.a	Nucleotides/ Modified Nucleotides
525	Malate	134	132.9	114.8	2.73	10	5	Neg	n.a	Organic acids
683	1,3-Diphosphoglycerate	266	265	79	0.87	37	5	Neg	n.a	Organic acids
964	Hydroxypyruvate	104.1	103	59	1.13	5	5	Neg	n.a	Organic acids
997	Phenylpyruvate	164.2	163	91	1.89	13	5	Neg	C00166	Organic acids
160419	Succinate	116.1	116.9	73	1.89	10	5	Pos	C00042	Organic acids
222656	(S)-Malate	134.1	133.1	115.1	6.34	10	5	Neg	n.a	Organic acids
444212	Aconitate	174.1	173	85	2.66	10	5	Neg	C00417	Organic acids
5460332	Linoleate	279.4	279.2	279.2	0.73	2	5	Neg	C01595	Organic acids
16219889	Phospho(enol) pyruvic acid	191	167	79	0.72	10	5	Pos	n.a	Organic acids
129669785	Phosphoglycerates	190	185	97	2.5	10	5	Neg	n.a	Organic acids
5460307	Fumarate	114.1	115	71	1.7	13	5	Neg	C00122	Organic acids
19	2,3-Dihydroxybenzoic acid	154.1	153	109	1.03	19	5	Neg	n.a	Organic acids

61	2,3-Diphosphoglyceric acid	266	265.1	167.1	3.27	14	5	Neg	n.a	Organic acids
77	2-Isopropylmalic acid	176.2	175.1	115	1.7	19	5	Neg	n.a	Organic acids
111	3-Ureidopropionate	132.1	133.1	90.1	6.25	10	5	Pos	C02642	Organic acids
119	4-Aminobutanoate	103.1	104.1	87.1	2.45	10	5	Pos	C00334	Organic acids
137	Aminolevulinic Acid	131.1	132.1	114.1	3.32	10	5	Pos	n.a	Organic acids
138	5-Aminopentanoate	117.2	118.2	55.1	3.46	16	5	Pos	C00431	Organic acids
338	Salicyclic acid (2-Hydrobenzoic acid)	183.1	137	93.2	1.37	12	5	Neg	n.a	Organic acids
385	Pimelic acid	160.1	158.8	96.8	2.74	12	5	Neg	n.a	Organic acids
464	Hippurate	179.2	178	77	1.28	20	5	Neg	C01586	Organic acids
469	Alpha-aminoadipic acid (a-AAA)	161.2	160.1	116	2.6	18	5	Neg	C00956	Organic acids
473	2-Oxo-4-methylthiobutanoate	148.2	147	99	3.63	13	5	Neg	n.a	Organic acids
487	2-Methylmalonate	118.1	117	73	1.9	4	5	Neg	C02170	Organic acids
487	Methylmalonic acid	118	116.8	73	2.8	8	5	Neg	C02170	Organic acids
500	4-Guanidinobutanoic acid	145.2	146.1	86	3.55	25	5	Pos	n.a	Organic acids
724	3-Phosphoglycerate	186.1	185	97	2.51	17	5	Neg	n.a	Organic acids
743	Glutaric acid	132.1	133.1	115.1	6.25	10	5	Pos	C00489	Organic acids
757	Glycolate	76.1	77.2	59.1	3.19	10	5	Pos	C00160	Organic acids
763	Guanidinoacetate	117.1	118.2	72.2	3.46	16	5	Pos	C00581	Organic acids
802	3-Indoleacetic acid	175.2	174	130.1	2.8	10	5	Neg	n.a	Organic acids
811	Itaconic acid	130.1	129	85	2.66	10	5	Neg	n.a	Organic acids
849	Pipecolic acid	129.2	130	84.1	3.36	18	5	Pos	C00408	Organic acids
867	Malonic acid	104	102.8	59	3.2	8	5	Neg	n.a	Organic acids
875	Tartaric acid	150.1	148.4	87	2.1	12	5	Neg	n.a	Organic acids
985	Palmitate	256.4	255.2	148.9	2.05	13	5	Neg	n.a	Organic acids
1018	Picolinic acid	123.1	124	78	1.62	16	5	Pos	n.a	Organic acids
1060	Pyruvic acid	88.1	88.7	43	2.8	10	5	Neg	n.a	Organic acids
1738	Homovanilic acid	182.2	181	137	2.03	10	5	Neg	n.a	Organic acids
3744	3-Indolepropionate	189.2	189.9	129.9	0.83	20	5	Pos	n.a	Organic acids
3848	3-Phenyllactic acid	166.2	164.8	146.9	1.12	10	5	Neg	n.a	Organic acids
4876	4-Aminobenzoate	136.1	138	94	3.17	15	5	Pos	n.a	Organic acids

5192	Sebacic acid	202.3	200.8	138.8	3.52	16	5	Neg	n.a	Organic acids
6119	2-Amino-2-methylpropanoate	103.1	104.1	45.1	2.5	10	5	Pos	C03665	Organic acids
6119	2-Aminoisobutyric acid	103.1	104	86	2.53	3	5	Pos	C03665	Organic acids
6723	4-Pyridoxate	183.2	182	138.2	1.75	10	5	Neg	C00847	Organic acids
6723	4-Pyridoxic acid	183.2	182.4	138.1	1.43	10	5	Neg	C00847	Organic acids
8742	Shikimate	174.2	173	93.1	1.25	18	5	Neg	C02637	Organic acids
10132	Mesoxalate	118	119.1	91.1	0.66	16	5	Pos	n.a	Organic acids
10457	Suberic acid	174.2	172.8	111	4.2	14	5	Neg	n.a	Organic acids
10972	Acetylglycine	117.1	118	76	4.37	10	5	Pos	n.a	Organic acids
11266	2-Hydroxybutyric acid	104.1	105.1	23.3	3.97	10	5	Pos	n.a	Organic acids
11671	2-hydroxyisobutyric acid	104.1	105.1	77	1.2	10	5	Pos	n.a	Organic acids
12033	4-Hydrobenzoic acid	183.1	137	93.2	1.37	12	5	Neg	n.a	Organic acids
12046	2-Methylglutaric acid	146.1	144.7	100.7	0.84	8	5	Neg	n.a	Organic acids
12121	3-Methylphenylacetic acid	150.2	149.002	105	0.66	12	5	Neg	n.a	Organic acids
12284	3-methylglutaric acid	146.1	144.7	100.7	0.84	8	5	Neg	n.a	Organic acids
16950	Argininosuccinate	290.3	289.1	271.1	1.68	10	5	Neg	C03406	Organic acids
64956	3-Aminoisobutanoate	103.1	104.2	86.1	2.5	10	5	Pos	C01205	Organic acids
69522	2-Aminooctanoic acid	159.2	160	55.3	2.79	21	5	Pos	n.a	Organic acids
71081	Oxalate	88	89	45	1.79	8	5	Pos	C00209	Organic acids
74563	2-Oxovalerate	116.1	117.1	43.1	3.35	10	5	Pos	C06255	Organic acids
90301	2,4-Dihydroxypyrimidine-5-carboxylic acid	156.1	157.1	139	3.21	12	5	Pos	n.a	Organic acids
91435	Lactate	89.1	89	43.2	1.81	16	5	Neg	C00186	Organic acids
92117	Homocysteic acid	183.2	184	138	3.43	10	5	Pos	n.a	Organic acids
93072	N-carbamoyl-L-aspartate	176.1	175	132	3.42	13	5	Neg	C00438	Organic acids
96215	4-Imidazoleacetic acid	126.1	127.2	109; 81	3.38	10	5	Pos	n.a	Organic acids
101122	2-Aminopimelic acid	175.2	174.1	130	2.8	12	5	Neg	n.a	Organic acids
123979	3-Carboxy-4-methyl-5-propyl-2-furanpropanoic acid (CMPF)	40.3	239	195	1	10	5	Neg	n.a	Organic acids
160617	Orotidylic acid	368.2	367	97	1.02	10	5	Neg	C01103	Organic acids
439194	D-Glyceric acid	106.1	107	79	2.67	10	5	Pos	n.a	Organic acids
439194	Glycerate	106.1	105	75	3.52	14	5	Neg	n.a	Organic acids

439774	3-Dehydroshikimate	172.1	171	127	1.51	10	5	Neg	n.a	Organic acids
444266	Maleic acid	116.1	115.03	71.03	1.25	13	5	Neg	n.a	Organic acids
487243	4-Aminopimelic acid	175.2	174.1	130	0.85	12	5	Neg	n.a	Organic acids
517460	2-Aminobutyrate	103.1	104.1	58.1	2.49	10	5	Pos	n.a	Organic acids
638129	Mesoconic	130.1	129	85	2.66	10	5	Neg	n.a	Organic acids
643798	Citraconic acid	130.1	129	85	2.66	10	5	Neg	n.a	Organic acids
1492348	Orotate	155.1	155	111	2.94	10	5	Neg	C00295	Organic acids
3037582	Galactaric acid	210.1	211.1	175.2	0.7	12	5	Pos	C00879	Organic acids
3541112	3-Hydroxybutyrate	103.1	103	59	1.13	5	5	Neg	n.a	Organic acids
3614358	Glyoxylate	73	73	45	3.3	8	5	Pos	C00048	Organic acids
5280498	Glutaconic acid	130.1	129	85	1.7	11	5	Neg	C02214	Organic acids
5280896	Abscisic acid	264.3	265	247.1	2.18	10	5	Pos	C06082	Organic acids
5375048	Indoleacrylic acid	187.2	186	142.03	1.91	20	5	Neg	n.a	Organic acids
5461056	Dihydroorotate	157.1	156.8	113	1.08	14	5	Neg	C00337	Organic acids
6419706	D-Gluconate	195.2	195	129	4.78	17	5	Neg	n.a	Organic acids
6971017	Acetoacetate	101.1	102.4	85	10	10	5	Pos	C00164	Organic acids
7018721	Ophthalmic acid	289.3	290.1	215	3.06	10	5	Pos	n.a	Organic acids
13943174	4-Aminoadipate	203.2	160.1	116	2.24	18	5	Neg	n.a	Organic acids
18998026	Aminoisobutyrate	103.1	104.1	86	2.47	16	5	Pos	C01205	Organic acids
54670067	Ascorbic acid	176.1	175.0	87	9.94	19	5	Neg	C01041	Organic acids
60150382	L-arginino-succinate	291.3	291	70	3.37	37	5	Pos	n.a	Organic acids
1176	Urea	60.1	61.1	44.2	1.87	25	5	Pos	C00086	Nitrogen metabolism
187	Acetylcholine	146.2	146.1	87	3.17	14	5	Pos	n.a	Choline metabolism
249	Betaine aldehyde	102.2	102	58	3.5	21	5	Pos	n.a	Betaine metabolism
438	1,3-Diaminopropane	74.1	75.1	43.2	1.08	10	5	Pos	n.a	Diamine biosynthesis
668	Dihydroxyacetone phosphate	170.1	18.2	165.1	0.77	10	5	Pos	n.a	Glycolytic pathway
700	Ethanolamine	61.1	62.2	44.2	1.79	12	5	Neg	C00189	Glycerophospholipid metabolism
753	Glycerol	92.1	93.1	61.1	2.71	10	5	Pos	n.a	Galactose/Glycerolipid metabolism
774	Histamine	111.2	112.1	95	6.04	15	5	Pos	n.a	Amino acid/Histidine metabolism
795	Imidazole	68.1	69	41.4	3.27	10	5	Pos	C01589	Histidine metabolism

798	Indole	117.2	118	91	2.85	26	5	Pos	C00463	Tryptophan metabolism
863	Lipoamide	205.3	206.1	189.1	2.65	10	5	Pos	C00579	Fatty amides/Fatty acyls
892	Myo-inositol	180.2	179	59.8	3.8	10	5	Neg	n.a	Carbohydrates/ Galactose metabolism
1030	Propylene glycol	76.1	77.1	59	3.37	10	5	Pos	C00583	Propanoate metabolism
1048	Pyrazole	68.1	68	41.2	3.47	19	5	Pos	C00481	Azole
1983	Acetaminophen	151.2	152	110	0.95	20	5	Pos	n.a	Anti-inflammatory
2153	Theophylline	180.2	181.1	124.1	0.98	19	5	Pos	C07130	Calcium signaling
2519	Caffeine	194.2	195	138	0.8	20	5	Pos	C07481	Alkaloids
4581	Octapamine	153.2	154.2	136.1	2.1	10	5	Pos	n.a	Phenylethanolamines
4687	Paraxanthine	180.2	181.2	124.1	0.98	21	5	Pos	C13747	Alkaloids
5429	Theobromine	180.2	181.2	138	1.01	17	5	Pos	C07480	Alkaloids
5570	Trigonelline	137.1	138.1	92.1	3.23	19	5	Pos	C01004	Alkaloids
5754	Cortisol	362.5	363.2	121	0.82	30	5	Pos	C00735	Steroid hormone biosynthesis
5780	Sorbitol	182.2	181.1	59	3.7	8	5	Neg	n.a	Fructose/ Galactose and mannose metabolism
5839	Aldosterone	360.4	359.2	189	0.73	24	5	Neg	C01780	Endocrine system
5994	Progesterone	314.5	313.2	276.8	3.4	25	5	Neg	C03207	Hormonal agent
6013	Testosterone	288.4	289.1	109	0.6	22	5	Pos	C00535	Steroid hormone biosynthesis
6251	Mannitol	182.2	181.1	59	3.7	8	5	Neg	n.a	Fructose and mannose metabolism
7027	Gluconolactone	178.1	177	99	3.63	10	5	Neg	C00198	Pentose phosphate pathway
7886	Phosphorylcholine	219.6	183	125	0.92	23	5	Pos	n.a	Glycerophospholipid metabolism
10111	Methylguanidine	73.1	74.2	42.2	2.95	10	5	Pos	C02294	Organonitrogen
10258	Indoxyl sulfate	213.2	134.2	106.2	0.88	15	5	Pos	n.a	Organic sulfuric acids and derivatives
10680	Flavone	222.2	223	121	3.27	29	5	Pos	n.a	flavonoids
10712	Cellobiose	342.3	341.1	263.1	0.91	14	5	Neg	n.a	Starch and sucrose metabolism
10935	Maleimide	97	98.2	70.1	2.58	15	5	Pos	C07272	Imide
11850	Galactitol	182.2	183.1	165.1	1.12	10	5	Pos	n.a	Galactose metabolism
12025	Pyrrolidinone	85.1	86.1	69.1	3.09	10	5	Pos	n.a	Nicotine degradation
24139	N-acetylglucosamine	221.2	220.1	58.7	1.55	18	5	Neg	n.a	Amino sugar and nucleotide sugar metabolism

65076	Cholesteryl sulfate	466.7	465.2	97	0.91	39	5	Neg	n.a	Steroid hormone biosynthesis
68400	Triuret	146.1	146.9	86.9	3.16	6	5	Pos	n.a	Purine metabolism
70120	6-Aminouracil	127.1	127.9	67.8	3	6	5	Pos	n.a	Methane/Riboflavin metabolism
99715	N-acetylasparagine	174.1	173.1	154.9	3.29	8	5	Neg; Pos	n.a	Modified amino acid
122357	D-Erythrose-4-phosphate	200.1	199	97	2.91	19	5	Neg	n.a	Pentose phosphate pathway
123658	D-ribulose 1,5-bisphosphate	310.1	308.9	97.1	0.85	19	5	Pos	n.a	Glyoxylate and dicarboxylate metabolism
123727	CDP-ethanolamine	446.2	445	273	0.69	29	5	Neg	n.a	Glycerophospholipid metabolism
164735	Sedoheptulose 1,7-bisphosphate	370.1	369	97	1.22	20	5	Neg	n.a	Carbon metabolism
171548	Biotin	244.3	245.1	227.1	2.27	10	5	Pos	C01893	Biotin metabolism
186078	Glycolaldehyde dimer	120.1	121.1	77.1	2.64	21	5	Pos	n.a	Glyoxylate and dicarboxylate metabolism
439162	Sn-glycerol-3-phosphate	172.1	171	79	4.91	15	5	Neg	n.a	Glycerolipid/ Glycerophospholipid metabolism
439168	D-Glyceraldehdye-3-phosphate	170.1	169.05	97	3.28	14	5	Neg	n.a	Glycolysis / Gluconeogenesis
439213	D-Glucosamine	179.2	180.2	163.1	3.23	10	5	Pos	n.a	Amino sugar and nucleotide sugar metabolism
439232	N-acetylornithine	174.2	175	115.1	6.06	16	5	Pos	n.a	Arginine biosynthesis
439918	N-amidino-L-aspartate	175.1	176.1	134	4.29	10	5	Pos	n.a	Amide/Amine
440049	Mannosamine	179.2	180.2	84	3.4	15	5	Pos	n.a	Amino sugar
2723790	Thiourea	76.1	77	60	3.21	10	5	Pos	C14415	Organosulfur compound
5280352	Bilirubin	584.7	583.3	285	0.68	36	5	Pos	C00486	Porphyrin metabolism
5281708	Daidzein	254.2	254.9	91	0.85	35	5	Pos	n.a	Isoflavonoid biosynthesis
10975657	Ribose	150.1	149	59.1	3.22	8	5	Neg	C00121	Pentose phosphate pathway
42609805	5-Phosphoribosyl-1-pyrophosphate	387.1	389	291	0.67	20	5	Neg	n.a	Pentose phosphate pathway
54598681	Cholesterol-3-sulfate	489.7	465.3	405.4	0.68	8	5	Neg	n.a	Steroid hormone biosynthesis
90657268	Octulose-monophosphate (O8P-O1P)	318.2	319	97	2.15	22	5	Neg	n.a	pentose phosphate pathway
125381971	3-Indoxyl-sulfate	255.3	212	80.3	1.7	20	5	Neg	n.a	Arylsulfates/Tryptophan pathway

129630434	N-acetylglucosamine phosphate	313.1	300	79	4.4	34	5	Neg	n.a	Amino sugar and nucleotide sugar metabolism
135398604	7,8-Dihydrofolate	443.4	444.2	178	1.83	32	5	Pos	n.a	Folate biosynthesis
135398752	Oxypurinol	152.1	153	136	1.12	20	5	Pos	n.a	Purine metabolism
135444742	Tetrahydrofolate	445.4	446.1	430.1	0.63	11	5	Pos	C00101	Glycine, serine and threonine metabolism
1045	Putrescine	88.2	89	72	4.92	12	5	Pos	n.a	Polyamine
1102	Spermidine	145.3	146.2	72	3.46	22	5	Pos	n.a	polyamine
1103	Spermine	202.3	202.1	129.1	1.18	19	5	Pos	n.a	polyamine
122356	Acetylputrescine	130.1	131.1	114.1	3.09	10	5	Pos	C02714	polyamine
203	Allantoic acid	176.1	177	61	2.74	10	5	Pos	n.a	Purine metabolites
204	Allantoin	158.1	157	114	2.74	10	3	Neg	C01551	Purine metabolites
1175	Uric acid	168.1	167	123.9	3.58	13	3	Neg	n.a	Purine metabolites
1188	Xanthine	152.1	151	108	2.1	18	5	Neg	C00385	Purine metabolites
190	Adenine	135.1	136	92	1.82	30	5	Pos	C00147	Purine metabolites
135398634	Guanine	151.1	152.2	123.9	0.15	16	5	Pos	n.a	Purine metabolites
135398635	Guanosine	283.2	284.1	135	0.65	35	5	Pos	n.a	Purine metabolites
135398638	Hypoxanthine	136.1	135	92	1.87	18	5	Neg	C00262	Purine metabolites
135398641	Inosine	268.2	269	137	2.04	5	5	Pos	C00294	Purine metabolites
104858	S-Nitrosoglutathione	336.3	337.1	307.1	0.72	5	5	Pos	n.a	Thiol metabolites
109	Cysteine-sulfinic acid	153.2	154.1	136	4.2	5	5	Pos	n.a	Thiol metabolites
595	Cystine	240.3	241.2	151.9	3.21	9	7	Pos	C00491	Thiol metabolites
834	Cystathionine	222.3	221.1	120.1	3.4	12	5	Pos	n.a	Thiol metabolites
847	Methionine sulfoxide	165.2	166.1	74	4.33	10	5	Pos	n.a	Thiol metabolites
975	Glutathione-oxidized (GSSG)	612.6	613.2	355.2	0.64	22	5	Pos	n.a	Thiol metabolites
1123	Taurine	125.2	124	80	4.19	18	5	Neg	C00245	Thiol metabolites
5862	Cysteine	121.2	122.1	75.9	4.93	12	5	Pos	C00097	Thiol metabolites
6137	Methionine	149.2	150.1	133	3.23	12	5	Pos	C00073	Thiol metabolites
10010	Homocystine	268.4	269.1	252.9	0.61	25	5	Pos	C01817	Thiol metabolites
15103	Selenomethionine	196.1	198.2	180.9	4.65	12	5	Neg	C05335	Thiol metabolites
16118	Methionine sulfoximine	180.2	181.1	74.1	3.44	15	5	Pos	n.a	Thiol metabolites

91552	Homocysteine	135.2	136.1	91	3.73	19	5	Pos	n.a	Thiol metabolites
107812	Hypotaurine	109.2	110.1	82.1	6	12	5	Pos	C00519	Thiol metabolites
134505	Homocysteine thiolactone	117.1	118.2	100	5.17	14	5	Pos	n.a	Thiol metabolites
134505	L-homocysteine thiolactone	117.2	118.2	100	6.17	14	5	Pos	n.a	Thiol metabolites
225710	Methylcysteine	135.2	136.02	119.02	1.58	12	5	Pos	n.a	Thiol metabolites
445282	Methionine sulfone	181.2	180.1	79.2	3.86	15	5	Neg	n.a	Thiol metabolites
448580	N-acetylmethionine	191.3	190.1	148	2.97	8	5	Neg	n.a	Thiol metabolites
131698098	Glutathione-reduced (GSH)	308.3	309.1	84	0.82	18	5	Pos	n.a	Thiol metabolites
65270	Cysteine-Glycine	178.2	179	76.2	6.01	15	5	Pos	n.a	Thiol metabolites
86	3-Hydroxyanthranilic acid	153.1	154.1	135.9	2.83	20	5	Pos	C19830	Tryptophan-Kynurenine metabolism
89	Hydroxykynurenine	224.2	225.1	208.1	4.6	10	5	Pos	C02794	Tryptophan-Kynurenine metabolism
846	Kynurenine	208.2	209.1	94.1	2.85	15	5	Pos	C00328	Tryptophan-Kynurenine metabolism
896	Melatonin	232.3	233.2	174.1	2.04	12	5	Pos	C01598	Tryptophan-Kynurenine metabolism
1826	5-Hydroxyindoleacetate	191.2	192.1	146	2.85	15	5	Pos	C05635	Tryptophan-Kynurenine metabolism
3845	Kynurenate	189.2	190.1	144.1	2.85	15	5	Pos	C01717	Tryptophan-Kynurenine metabolism
5202	Serotonin	176.2	177.1	160.1	3.11	20	5	Pos	n.a	Tryptophan-Kynurenine metabolism
5699	Xanthurenic acid	205.2	204	160	3	19	5	Pos	n.a	Tryptophan-Kynurenine metabolism
6305	Tryptophan	204.2	205.1	188.1	2.86	10	5	Pos	C00078	Tryptophan-Kynurenine metabolism
69867	Indole-3-carboxylic acid	161.2	160.002	116	2.52	20	5	Neg	n.a	Tryptophan-Kynurenine metabolism
70547	N-acetyltryptamine	202.3	203.1	144.2	2.4	15	5	Pos	n.a	Tryptophan-Kynurenine metabolism
5459842	Anthranilate	136.1	136	92	2.17	18	5	Neg	n.a	Tryptophan-Kynurenine metabolism
1066	Quinolinate	167.1	168	124	2.92	15	5	Pos	n.a	Tryptophan-Kynurenine metabolism
1130	Thiamine (Vitamin B1)	265.4	265	122	2.68	19	5	Pos	C00378	Vitamins
14985	Alpha-Tocopherol (Vitamin E)	430.7	431.4	165	0.61	17	5	Pos	C02477	Vitamins
1052	Pyridoxamine (Vitamin B6)	168.2	169	152	3.34	11	5	Pos	C00534	Vitamins
1054	Pyridoxine (Vitamin B6)	169.2	170	134	0.82	18	5	Pos	C00314	Vitamins
6613	Pantothenic acid (Vitamin B5)	219.2	218	146	1.45	21	5	Neg	C00864	Vitamins

493570	Riboflavin (Vitamin B2)	376.4	375.1	254.9	1.70	15	5	Neg	n.a	Vitamins
5280793	Vitamin D2	396.6	397.3	397.3	0.71	5	5	Pos	n.a	Vitamins
5280795	Vitamin D3	384.6	385.3	367.2	0.75	5	5	Pos	C01164	Vitamins
5284607	Vitamin K1	450.7	451.4	187	0.65	25	5	Pos	C03313	Vitamins
**Vendor**	**Commercial name***									
Cayman Chemical	7α-Hydroxy-4-cholesten-3-one-d7 (7-HCO-D7)	407.68	408.1	115.1	6.34	10	5	Pos	n.a	Internal standard
Cayman Chemical	7α,12α-Dihydroxycholest-4-en-3-one-d7 (7,12-DHCO-D7)	423.68	425.2	389.3	0.705	15	5	Pos	n.a	Internal standard
Cayman Chemical	8 Isoprostane-D11	365.6	357.4	43.2	1.08	10	5	Pos	n.a	Internal standard
Sigma-Merck	ADMA -D6	281.21	210.4	79	0.87	37	5	Neg	n.a	Internal standard
Sigma-Merck	Allantoin-13C2,15N4	164.07	162.9	85	2.26	15	5	Pos	n.a	Internal standard
Sigma-Merck	Arginine·HCl [13C6, 99%; 15N4, 99%]	185.2	185.1	85	0.93	15	5	Pos	n.a	Internal standard
Sigma-Merck	Aspartic acid [13C4, 99%; 15N, 99%]	139.1	139.1	92.1	2.7	12	5	Pos	n.a	Internal standard
Sigma-Merck	L-Carnitine:HCl, O-Butyryl (N-Methyl-D3, 98%)	270.77	235.1	124.1	5.54	14	5	Pos	n.a	Internal standard
Sigma-Merck	Cystine [13C6, 99%; 15N2, 99%]	155.1	249	93.1	3.34	21	5	Pos	n.a	Internal standard
Sigma-Merck	Creatinine-D3	116.14	117.1	47.1	2.5	19	5	Pos	n.a	Internal standard
Cambridge Isotopes laboratories	Chenodeoxycholic acid (CDCA) (2,2,3,4,4-D5, 98%) CDCA-D5	397.6	395.3	395.3	0.857	30	5	Neg	n.a	Internal standard
Cambridge Isotopes laboratories	Deoxycholic acid (DCA) (2,2,4,4-D4, 98%),	396.6	395.3	395.3	0.857	30	5	Neg	n.a	Internal standard
Cambridge Isotopes laboratories	Glycochenodeoxycholic acid (GCDCA) (2,2,4,4-D4, 98%),	458.68	448.3	74	0.95	10	5	Neg	n.a	Internal standard
Cambridge Isotopes laboratories	Glycodeoxycholic acid (GDCA) (2,2,4,4-D4, 98%)	453.65	448.3	74	0.95	10	5	Neg	n.a	Internal standard
Cambridge Isotopes laboratories	Taurodeoxycholic acid, (TDCA) (2,2,4,4,11,11-D6, 98%)	527.7	395.3	395.3	1..854	30	5	Neg	n.a	Internal standard

Cambridge Isotopes laboratories	Lithocholic acid (LCA)(2,2,4,4-D4, 98%),	380.6	379.3	379.3	0.736	30	5	Neg	n.a	Internal standard
Cambridge Isotopes laboratories	Glycourseodeoxycholic acid (GUDCA) (2,2,4,4-D4, 98%)	453.65	448.3	74	0.95	10	5	Neg	n.a	Internal standard
Tetrahedron	L-Ergothioneine-D9	238.1	239.3	109	1.03	19	5	Neg	n.a	Internal standard
Cambridge Isotopes laboratories	Glycocholic acid (GCA) (2,2,4,4-D4, 98%)	469.65	468.3	74	1.2	70	5	Neg	n.a	Internal standard
Cambridge Isotopes laboratories	Glycochenodeoxycholic acid (GCDCA) (2,2,4,4-D4, 98%)	453.65	452.3	74	1.31	72	5	Neg	n.a	Internal standard
Sigma-Merck	L-Glutamic acid [13C5, 99%; 15N, 99%]	155.1	154.1	167.1	3.27	14	5	Neg	n.a	Internal standard
Sigma-Merck	L-Glutamine [13C5, 99%]	152.1	152.1	139	3.21	12	5	Pos	n.a	Internal standard
Tetrahedron	L-Hercynine -D9	206.5	207.2	85	0.93	25	5	Pos	n.a	Internal standard
Sigma-Merck	L-Histidine·HCl·H2O [13C6, 97-99%; 15N3, 97-99%]	164.2	165.2	118.2	5.16	15	5	Pos	n.a	Internal standard
Sigma-Merck	DL-Homocysteine (3,3,4,4-D4, 98%)	139.21	140.2	85	0.736	25	5	Pos	n.a	Internal standard
Sigma-Merck	Inosine (15N4, 95%+)	272.2	271.1	139.1	2.856	15	5	Neg	n.a	Internal standard
Sigma-Merck	L-Isoleucine (13C6, 99%; 15N, 99%)	139.2	139	45.1	2.5	10	5	Pos	n.a	Internal standard
Sigma-Merck	Kynurenic acid (Ring-D5, 98%)	194.2	195.2	149.2	2.362	18	5	Pos	n.a	Internal standard
Sigma-Merck	L-leucine [13C6, 99%; 15N, 99%]	139.2	138.9	55.3	2.79	21	5	Pos	n.a	Internal standard
Sigma-Merck	L-Lysine·2HCl [13C6, 99%; 15N2, 99%]	155.2	155.2	130	2.8	12	5	Neg	n.a	Internal standard
Sigma-Merck	L-Methionine [13C5, 99%; 15N, 99%]	156.2	156.2	113	3.4	10	5	Pos	n.a	Internal standard
Sigma-Merck	Vitamin B3 (Nicotinic acid) (D4, 98%)	127.13	128.1	84.1	1.18	24	5	Pos	n.a	Internal standard
Sigma-Merck	Propionyl-L-carnitine-d3 HCl (N-methyl-D3)	256.74	221.2	115	1.7	19	5	Neg	n.a	Internal standard
Sigma-Merck	L-Serine [13C3, 99%; 15N, 99%]	110.1	110.2	73	1.901	4	5	Neg	n.a	Internal standard
Sigma-Merck	Spermidine -3HCl (13C4, 99%)	258.6	150.2	99	3.63	13	5	Neg	n.a	Internal standard
Sigma-Merck	L-Threonine [13C4, 97-99%; 15N, 97-99%]	125.1	125.1	43.1	3.35	10	5	Pos	n.a	Internal standard
Sigma-Merck	L-Tyrosine [13C9, 99%; 15N, 99%]	192.2	192.1	170	3.23	12	5	Pos	n.a	Internal standard
Sigma-Merck	L-Tryptophan (1-13C, 99%)	205.22	206.4	189.4	3.02	9	5	Pos	n.a	Internal standard

Sigma-Merck	Uric acid-1,3-15N2	170.1	169	181.1	3.63	12	5	Neg	n.a	Internal standard
Sigma-Merck	Valine 13C5, 99%; 15N, 99%	124.1	123.1	86.1	2.5	10	5	Neg	n.a	Internal standard
Sigma-Merck	Xanthine (1,3-15N2, 98%+) 90% Pure	154.1	153.1	127	1.509	10	5	Neg	n.a	Internal standard
Sigma-Merck	L-Phenylalanine [13C9, 99%; 15N, 99%]	176.2	176.1	129.1	2.3	10	5	Pos	n.a	Internal standard
Sigma-Merck	L-Proline [13C5, 99%; 15N, 99%]	122.1	122.1	75.1	3.41	14	5	Pos	n.a	Internal standard

* There are no pubChem ID for these stable isotope internal standards, MW = molecular weight, n.a = not available in current database

Supplemental Table 3. Multiplex oxidative stress panel components visualized by volcano plot comparing sepsis vs control groups

Metabolite	FC	log2(FC)	P-value [a]
Lysine	0.60	-0.75	3.97E-24
Glutamine	0.61	-0.72	1.33E-21
hArg	0.45	-1.14	5.14E-16
Arg	0.59	-0.77	7.59E-16
LysoPC 20:0	0.49	-1.03	3.69E-15
Citrulline	0.58	-0.79	3.82E-15
LysoPC 20:3	0.47	-1.08	6.08E-15
Acetylornithine	0.58	-0.79	6.35E-15
N-methylnicotinamide	0.41	-1.28	9.45E-15
Methylnicotinamide	0.41	-1.28	9.45E-15
Glycerophosphocholine	0.55	-0.87	9.45E-15
N6-acetyllysine	0.44	-1.17	5.55E-14
N-acetyl-galactosamine	2.20	1.14	6.16E-14
Acetylglycine	0.52	-0.93	6.16E-14
Uracil	0.54	-0.89	1.19E-13
Trans-4-hydroxyproline	0.56	-0.83	1.22E-13
LysoPC 22:6	0.54	-0.90	1.12E-12
LysoPC 20:4	0.53	-0.91	1.19E-12
5 oxo-d proline	0.75	-0.42	4.34E-11
Uridine	0.56	-0.83	5.25E-11
5-HTP	0.66	-0.59	9.21E-11
Pyrrolidinone	0.63	-0.66	1.17E-10
2,3-dihydroxybenzoic acid	0.70	-0.51	2.23E-10
Acetyl-dl-carnitine	2.29	1.19	3.09E-10
Hexanoylcarnitine	2.29	1.19	3.09E-10
Methionine sulfoxide	1.75	0.81	3.34E-10
Acetylputrescine	0.64	-0.64	5.31E-10
Thymine	0.69	-0.53	5.87E-10
Sarcosine	0.67	-0.59	6.18E-10
N-acetyl-glutamine	1.85	0.89	9.09E-10
Phenylalanine	1.28	0.35	1.48E-09
Adipylcarnitine	4.78	2.26	1.85E-09
Tiuret	0.76	-0.39	1.85E-09
Choline	0.74	-0.44	2.29E-09
5:1 carnitine	2.37	1.25	4.52E-09
Aminoisobutyrate	0.71	-0.50	4.61E-09
C4-OH	3.51	1.81	5.86E-09
Malonylcarnitine	3.37	1.75	5.86E-09
Oxaloacetate	1.77	0.82	6.32E-09
N-alpha-acetyl-L-asparagine	0.76	-0.40	1.14E-08
Cyanobalmin	0.51	-0.98	1.37E-08
Isoleucine	0.75	-0.42	1.46E-08
Deoxyadenosine	0.71	-0.48	3.30E-08
Hydroxydodecenoylcarnitine	2.02	1.02	3.37E-08

Proline	0.77	-0.38	5.24E-08
Mesoxalate	0.65	-0.61	5.98E-08
N-acetyl-DL-glutamic acid	1.74	0.80	5.98E-08
LysoPC 20:1	0.61	-0.72	7.34E-08
Methionine	0.77	-0.38	9.58E-08
SDMA	2.03	1.02	1.01E-07
Betaine	0.76	-0.39	1.02E-07
Betaine aldehyde	0.74	-0.44	1.68E-07
Methionine sulfoxide	1.76	0.81	1.87E-07
L-ornithine	0.75	-0.42	2.43E-07
LysoPC 20:5	0.63	-0.68	2.66E-07
Alanine	0.73	-0.45	3.10E-07
Creatinine	1.78	0.83	3.55E-07
Dimethylglycine	0.74	-0.44	3.82E-07
Epinephrine	0.78	-0.36	3.82E-07
Imidazole	0.64	-0.65	4.69E-07
C5:1	2.07	1.05	4.80E-07
N-acetylglycine	0.71	-0.50	7.03E-07
3-aminoisobutanoate	0.75	-0.42	8.48E-07
Purine	1.47	0.56	8.74E-07
(S)-malate	0.75	-0.41	9.17E-07
Glutaric acid	0.75	-0.41	9.17E-07
Histamine	0.79	-0.35	9.65E-07
Hypotaurine	0.78	-0.36	1.08E-06
N-acetyl-glutamine	0.71	-0.50	1.33E-06
Histidinol	3.55	1.83	1.48E-06
Tryptophan	0.70	-0.52	1.53E-06
Allantoin	2.12	1.08	1.64E-06
17a-Hydroxyprogesterone	1.41	0.49	2.11E-06
Pyrazole	0.77	-0.39	2.96E-06
L-kynurenine	1.77	0.82	3.86E-06
Butyryl carnithine	1.63	0.70	3.86E-06
N-amidino-aspartate	3.16	1.66	3.89E-06
DL-Pipecolic acid	0.70	-0.51	3.94E-06
N-acetylglycine	0.69	-0.54	4.49E-06
Urea	1.53	0.61	1.14E-05
L-norvaline	0.79	-0.34	1.16E-05
O-acetyl-L-serine	1.82	0.86	1.34E-05
Biotin	2.85	1.51	1.45E-05
Valine	0.80	-0.32	1.49E-05
Pipecolate	0.52	-0.94	1.66E-05
5-hydroxyindoleacetate	1.65	0.72	1.76E-05
Asparagine	0.84	-0.26	2.68E-05
Indole	0.74	-0.43	2.78E-05
Pyridoxamine	1.26	0.34	2.82E-05
Kynurenate	0.69	-0.53	3.03E-05
Guanosine	1.50	0.58	4.01E-05
Cholic acid	0.79	-0.34	4.34E-05

5-aminopentanoate	0.81	-0.31	4.58E-05
7-HCO	0.57	-0.81	5.30E-05
Hydroxypyruvate	2.69	1.43	7.12E-05
Cis-4-hydroxyproline	0.82	-0.28	1.71E-04
Xanthine/oxypurinol	2.29	1.20	1.72E-04
Valerylcarnitine	1.43	0.52	1.93E-04
1-methyl-histidine	1.82	0.86	2.55E-04
Glycine	3.34	1.74	2.65E-04
Cystine	1.68	0.75	3.82E-04
Creatine phosphate	1.56	0.64	4.15E-04
Argininosuccinate	1.86	0.90	4.94E-04
A-Tocopherol	1.34	0.42	5.05E-04
N6,N6,N6-Trimethyl-L-lysine	1.55	0.63	5.16E-04
Inosine	0.89	-0.16	6.31E-04
Progesterone	0.75	-0.42	6.45E-04
Tyrosine	0.77	-0.38	6.69E-04
Vitamin K1	0.70	-0.52	7.21E-04
LysoPC 24:0	0.68	-0.55	7.31E-04
Imidazolaeacetic acid	2.20	1.14	8.07E-04
Succinate	1.48	0.57	9.62E-04
N-acetyl-asparagine	0.88	-0.19	9.78E-04
3-chlorotyrosine	0.74	-0.43	9.91E-04

P-values [a] were determined by both fold change (FC) and unpaired t-test with Bonferroni correction. $P < 0.05$ is statistically significant. The log2 scale of the fold change was used to compare changes in both directions equidistant from the baseline zero.

Supplemental Table 4. Multiplex oxidative stress panel components visualized by volcano plot comparing sepsis vs infection groups

Metabolite	FC	$Log_2(FC)$	P-value [a]
SDMA	2.122	1.0854	4.28E-20
1-Methyl-Histidine	2.264	1.1789	5.76E-20
Creatinine	1.7291	0.79003	6.04E-19
O-Adipoyl-L-Carnitine	4.5484	2.1853	8.95E-18
N-acetyl-glutamine	1.7769	0.82938	9.32E-17
Noradrenaline	2.2085	1.143	2.54E-16
O-Tiglylcarnitine	2.2899	1.1953	1.21E-15
NMMA	1.857	0.89296	5.29E-14
ADMA	1.821	0.86473	6.03E-14
Biotin	2.9361	1.5539	9.31E-14
N-acetyl-DL-glutamic acid	1.6527	0.72481	2.43E-12
4-hydroxy-L-proline	1.5855	0.66491	9.00E-12
Oxaloacetate	1.9279	0.94701	5.61E-11
Acetyl-DL-Carnitine	1.8446	0.88333	5.61E-11
Hexanoylcarnitine	1.8446	0.88333	5.61E-11
Hydroxydodecenoylcarnitine	1.7996	0.8477	5.61E-11
Nicotinic Acid	1.754	0.81064	8.32E-11
butyryl carnithine	1.6558	0.72752	8.59E-11
L-kynurenine	1.6943	0.76066	1.20E-10
O-Glutarylcarnitine	5.559	2.4748	2.75E-10
Hydroxyhexanoycarnitine	5.559	2.4748	2.75E-10
5-hydroxyindoleacetate	1.6082	0.68549	4.65E-10
Ethanolamine	1.4258	0.51178	6.29E-09
Oxypurinol	3.8952	1.9617	6.76E-09
Cystine	1.7357	0.79555	1.08E-08
7-methylguanosine	2.0355	1.0254	1.49E-08
5-methylcytosine hydrochloride	2.896	1.5341	2.83E-08
LysoPC 20:3	0.62567	-0.67653	4.90E-08
Succinate	1.583	0.6627	5.36E-08
Creatine Phosphate	1.5928	0.67154	1.13E-07
17a hydroxyprogesteron	1.3118	0.39154	3.16E-07
Abscisic Acid	10.083	3.3339	4.48E-07
Succinylcarnitine	1.4693	0.5551	5.13E-07
Glycine	3.0177	1.5934	5.40E-07
Trans-3-Hexenoic Acid	1.5552	0.63714	5.61E-07
selenomethionine	2.061	1.0433	7.98E-07
C4-dicarboxylate	1.4849	0.57041	1.02E-06
3-hydroxy-isovalerylcarnitine	1.4849	0.57041	1.02E-06
Urea	1.3571	0.44058	1.02E-06
Thiourea	3.2106	1.6829	1.68E-06
N-acetyl-L-aspartic acid	2.2992	1.2011	1.93E-06
Acetylputrescine	0.82817	-0.27199	2.31E-06
Methionine sulfoxide	1.4706	0.55637	4.36E-06
Methyl-Histidines	3.0788	1.6224	8.86E-06

Trimethylamine N-oxide	2.2785	1.1881	8.86E-06
Melatonin	1.8692	0.9024	1.76E-05
N-carbamoyl-L-aspartate	1.3993	0.48468	2.15E-05
Ribose	3.8053	1.928	2.90E-05
2,3-dihydroxybenzoic acid	0.85985	-0.21784	4.37E-05
L-DOPA	1.4995	0.58449	5.47E-05
Trimethylamine	2.1092	1.0767	6.18E-05
Malonylcarnitine	1.9659	0.97516	6.18E-05
Hydroxybutyrylcarnitine	2.0124	1.0089	6.27E-05
Phenylalanine	1.1352	0.18291	6.27E-05
3-methylhistamine	2.7866	1.4785	6.73E-05
LysoPC 20:4	0.71844	-0.47706	6.81E-05
N-Acetylisoleucine	2.3767	1.249	7.30E-05
Creatine	1.309	0.38841	7.86E-05

P-value [a] was determined by both fold change (FC) and unpaired t-test with Bonferroni correction. $P < 0.05$ is statistically significant. The log2 scale of the fold change was used to compare changes in both directions equidistant from the baseline zero.

Supplemental Table 5. Significant plasma metabolites identified by volcano analysis comparing sepsis vs control groups and sepsis vs infection groups

Sepsis vs control				Sepsis vs infection			
**Metabolite**	**Fold Change**	**log_2(FC)**	**P-value [a]**	**Metabolite**	**Fold Change**	**log_2(FC)**	**P-value [b]**
hArg	0.45	-1.1424	5.14E-16	SDMA	2.12	1.0854	4.28E-20
Lyso PC 20:0	0.48	-1.0325	3.69E-15	1-methyl-histidine	2.26	1.1789	5.76E-20
Lyso PC 20:3	0.47	-1.0752	6.08E-15	O-adipoyl-l-carnitine	4.54	2.1853	8.95E-18
N-Methylnicotinamide	0.41	-1.2824	9.45E-15	Noradrenaline	2.20	1.143	2.54E-16
N6-acetyllysine	0.44	-1.1743	5.55E-14	O-tiglylcarnitine	2.28	1.1953	1.21E-15
N-acetyl galactosamine	2.2	1.1397	6.16E-14	Biotin	2.93	1.5539	9.31E-14
Acetyl-DL-Carnitine	2.28	1.1931	3.09E-10	O-glutarylcarnitine	5.5	2.4748	2.75E-10
Hexanoylcarnitine	2.28	1.1931	3.09E-10	Hydroxyhexanoycarnitine	5.55	2.4748	2.75E-10
O-adipoylcarnitine	4.77	2.2563	1.85E-09	Oxypurinol	3.89	1.9617	6.76E-09
O-tiglylcarnitine	2.37	1.2451	4.52E-09	7-methylguanosine	2.03	1.0254	1.49E-08
Hydroxybutyrylcarnitine	3.51	1.8134	5.86E-09	5-methylcytosine Hydrochloride	2.89	1.5341	2.83E-08
Malonylcarnitine	3.37	1.7514	5.86E-09	Abscisic acid	10.08	3.3339	4.48E-07
Hydroxydodecenoylcarnitine	2.02	1.0165	3.37E-08	Glycine	3.01	1.5934	5.40E-07
SDMA	2.02	1.0209	1.01E-07	Selenomethionine	2.06	1.0433	7.98E-07
(Z)-pent-3-enoic acid	2.07	1.052	4.80E-07	Thiourea	3.21	1.6829	1.68E-06
Histidinol	3.55	1.8281	1.48E-06	N-acetyl-L-aspartic acid	2.29	1.2011	1.93E-06
Allantoin	2.12	1.0843	1.64E-06	TMAO	2.27	1.1881	8.86E-06
N-amidino-aspartate	3.15	1.6578	3.89E-06	Ribose	3.80	1.928	2.90E-05
Biotin	2.84	1.5088	1.45E-05	TMA	2.10	1.0767	6.18E-05
Hydroxypyruvate	2.69	1.4295	7.12E-05	Hydroxybutyrylcarnitine	2.01	1.0089	6.27E-05
				3-methylhistamine	2.78	1.4785	6.73E-05
				CMPF	2.37	1.249	7.30E-05

P-value [a] was determined by both fold change (FC) and unpaired t-test with Bonferroni correction comparing sepsis vs control groups, and P-value [b] sepsis vs infection groups. $P < 0.05$ is statistically significant. The log2 scale of the fold change was used to compare changes in both directions equidistant from the baseline zero.

Supplemental Table 6. Linear regression model of hArg and methylarginines (ADMA, SDMA, NMMA) for diastolic arterial pressure

Linear regression	Arg		hArg		hArg:SDMA		hArg:ADMA		hArg:NMMA	
	β [95% CI]	P value	β [95% CI]	P value	β [95% CI]	P value	β [95% CI]	P value	β [95% CI]	P value
Univariate [a]	8.68 [2.87-14.50]	0.004	6.98 [2.81-11.15]	0.001	7.10 [3.91-10.29]	<0.001	6.85 [2.52-11.18]	0.002	7.67 [3.58-11.77]	<0.001
Multivariate [b]	8.86 [3.03-14.69] [b]	0.003	6.54 [2.19-10.88] [b]	0.003	6.63 [2.71-10.56] [b]	<0.001	5.71 [1.12-10.31] [b]	0.015	6.38 [2.01-10.76] [b]	0.004

Linear regression model using forward method and P-value and regression coefficients (β) in univariable **[a]** and multivariable **[b]** analysis. Linear regression coefficient (β [95% CI] [b] adjusted for age, gender, creatinine, and comorbidities. $P < 0.05$ is statistically significant. Abbreviations: Unstandardized Coefficients (β). 95% confidence interval (95% CI). (hArg:ADMA) plasma hArg/ADMA ratio, (hArg:SDMA) plasma hArg/SDMA ratio, and (hArg:NMMA) plasma hArg/NMMA ratio.

Supplemental Table 7. Z test for pairwise comparison of ROC AUC performance for ICU admission

Pair(s) result	z	P-value	AUC Difference [95% CI]
SOFA vs. metabolite model	1.100	0.271	0.05 [-0.04-0.14]
SOFA vs. combined model	-1.121	0.262	-0.03 [-0.08-0.02]
metabolite model vs. combined model	-2.632	0.008	-0.08 [-0.14-0.02]

Pairwise comparisons of the ROC AUC of SOFA model, metabolite model and combined model (SOFA + metabolite model) for ICU admission. Each model was adjusted for age, gender, creatinine, and comorbidities. $P < 0.05$ is statistically significant. SOFA vs. metabolite model (Z = 1.100, 0.271), SOFA vs. combined model (Z = -1.121, 0.262), and metabolite model vs. combined model (z = -2.632, P = 0.008).

Supplemental Fig. 1 Quality Control and Analysis Plan.

A.

				number of analytes
Data Generation	Phase 0	Data acquisition	Extraction protocol, column selection, machine settings	-
	Phase 1	Data processing	The unique analyte list includes all measured entities	385
			Analytes with isotopic standards	38
			For analytes without isotopic standards	297
			- if part of a structural class, then use related ISTD	297
			- if not part of a structural class, then no ISTD is used	0
			Peaks are integrated (automated + manual review)	352
			Batch correction is applied	335
	Phase 1a	Analyte inclusion	Accept if	
			- CoV of analyte repeat measurement in QC sample < 30%	158
			or	
			- Mean analyte D-ratio across study samples < 50%	246
			Formation of the processed analyte list	269

B.

Data Analysis	Phase 2	Dimensionality reduction and selection of interesting analytes	Data transformation and scaling	269
			Pairwise fold change and p-value ranking	269
			Correlation matrix	269
			PCA / PLS-DA VIP ranking	269
			Interesting analytes (NO pathway panel)*	19
			PLS-DA VIP	19
			Multivariable regression	5
			ROC curve ranking	5
			Clinical candidate list (hArg)	1

The selection of 'interesting analytes' list is based on a manual inspection of VIP rank, fold-change, p-value of group differences and frequency of pathway appearance as a class in ranked analyses. It is a combination of objective measures and a subjective judgement their review.

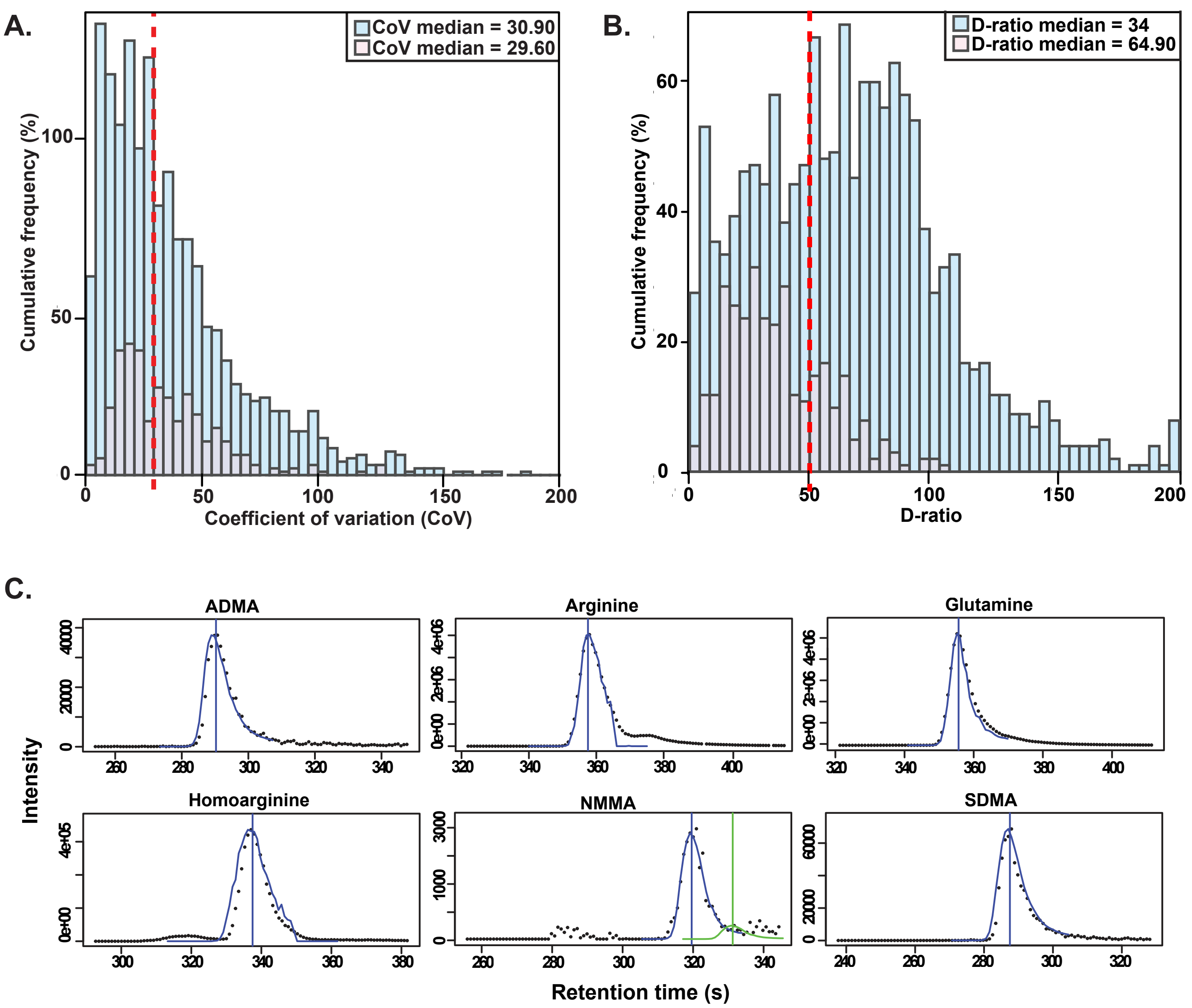

Supplemental Fig. 2 Quality Control and Analysis Plan. The selection of 'interesting analytes' list is based on a manual inspection of VIP rank, fold-change, p-value of group differences and frequency of pathway appearance as a class in ranked analyses. It is a combination of objective measures and a subjective judgement their review.

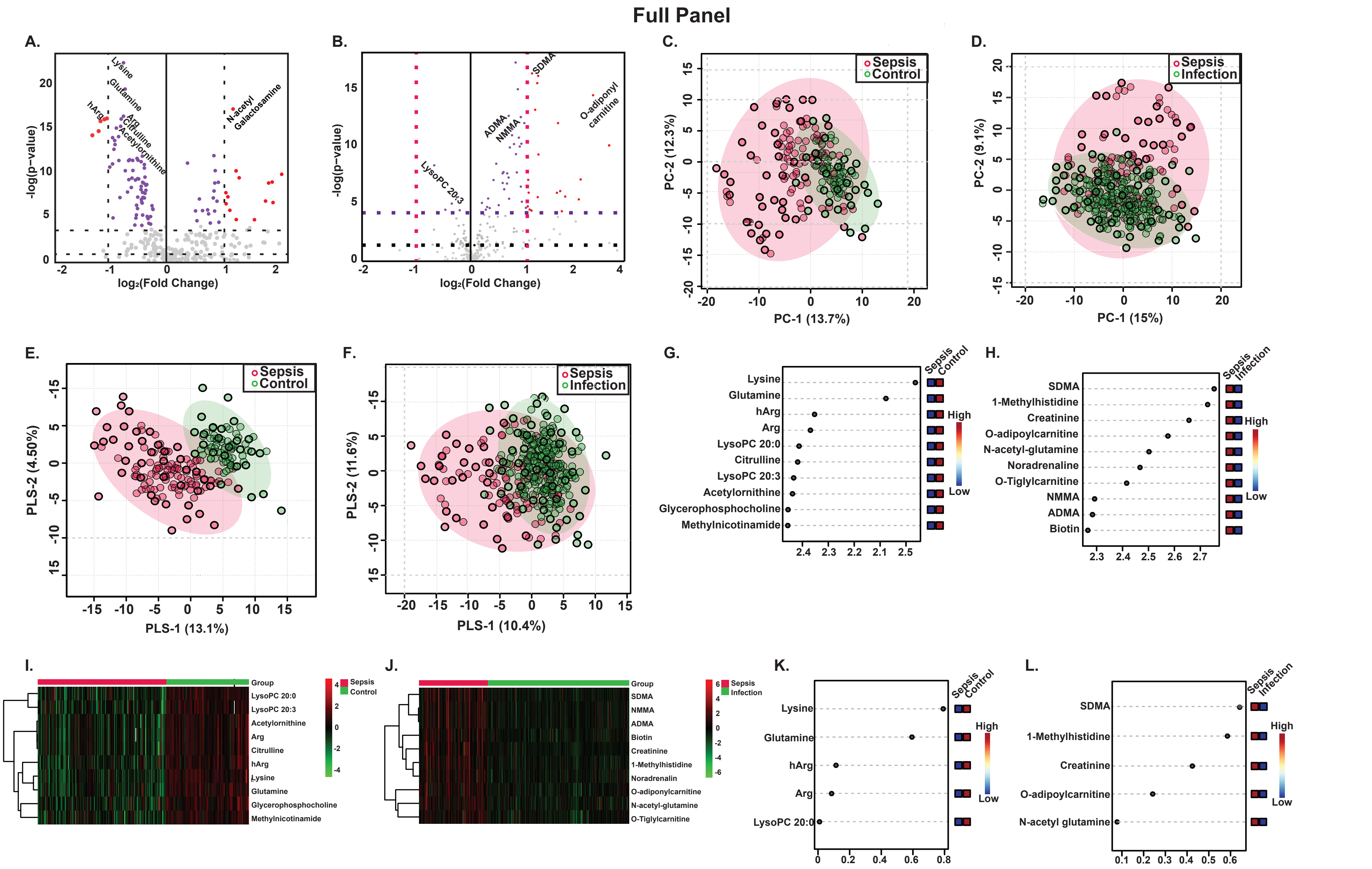

Supplemental Fig. 3 Full panel analysis and interrogation of 269 metabolites identified major metabolites associated with sepsis. A. Volcano plot of metabolites comparing sepsis vs control groups and **B.** sepsis vs infection groups. Analytes met stringent criteria (fold change, FC > 2, adjusted P-value < 0.0001) are marked in red circles. **B-C.** Initial screening by PCA plots for separation of **C.** sepsis vs control groups, and **d.** sepsis vs. infection groups. **E-F.** Supervised partial least square discriminant analysis (PLS-DA) discriminating **E.** sepsis vs control groups and **F.** sepsis vs infection groups, and **G-H.** identification of key features by the model (VIP score > 2) for **G.** sepsis vs control and **H.** sepsis vs infection. Important features were ranked by PLS-DA based on variable importance in projection (VIP) score on the x-axis and significant metabolites (VIP score >2.0) on the y-axis. **I-J.** Cluster heatmap shows differential expression of predominant metabolic biomarkers between **I.** sepsis vs control groups and **J.** sepsis vs infection groups. **K-L.** sparse partial least square discriminant analysis (sparse PLS-DA) further selected the most significant features associated with sepsis.

NO Pathway Panel

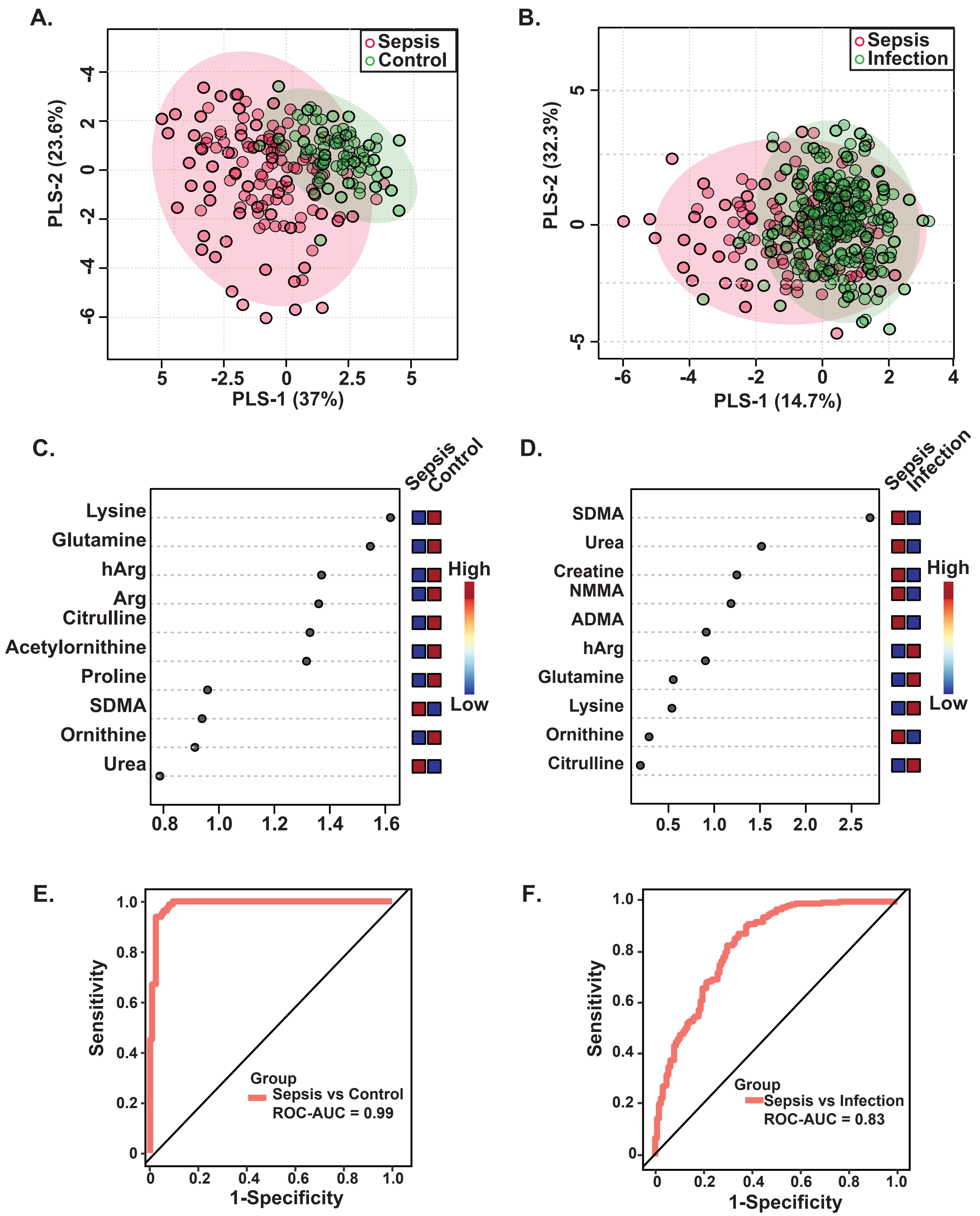

Supplemental Fig. 4 Segmental analysis targeted 19 metabolites in the arginine-NO pathway identified the most significant metabolites in sepsis. A-B. PLS-DA plots for **A.** sepsis vs control and **B.** sepsis vs infection groups. **C-D.** Key features identified by PLS-DA (VIP score > 1). **E-F.** ROC AUC for PLS-DA VIP-features shows high accuracy of each model for discriminating **E.** sepsis vs control and **F.** sepsis vs infection groups.

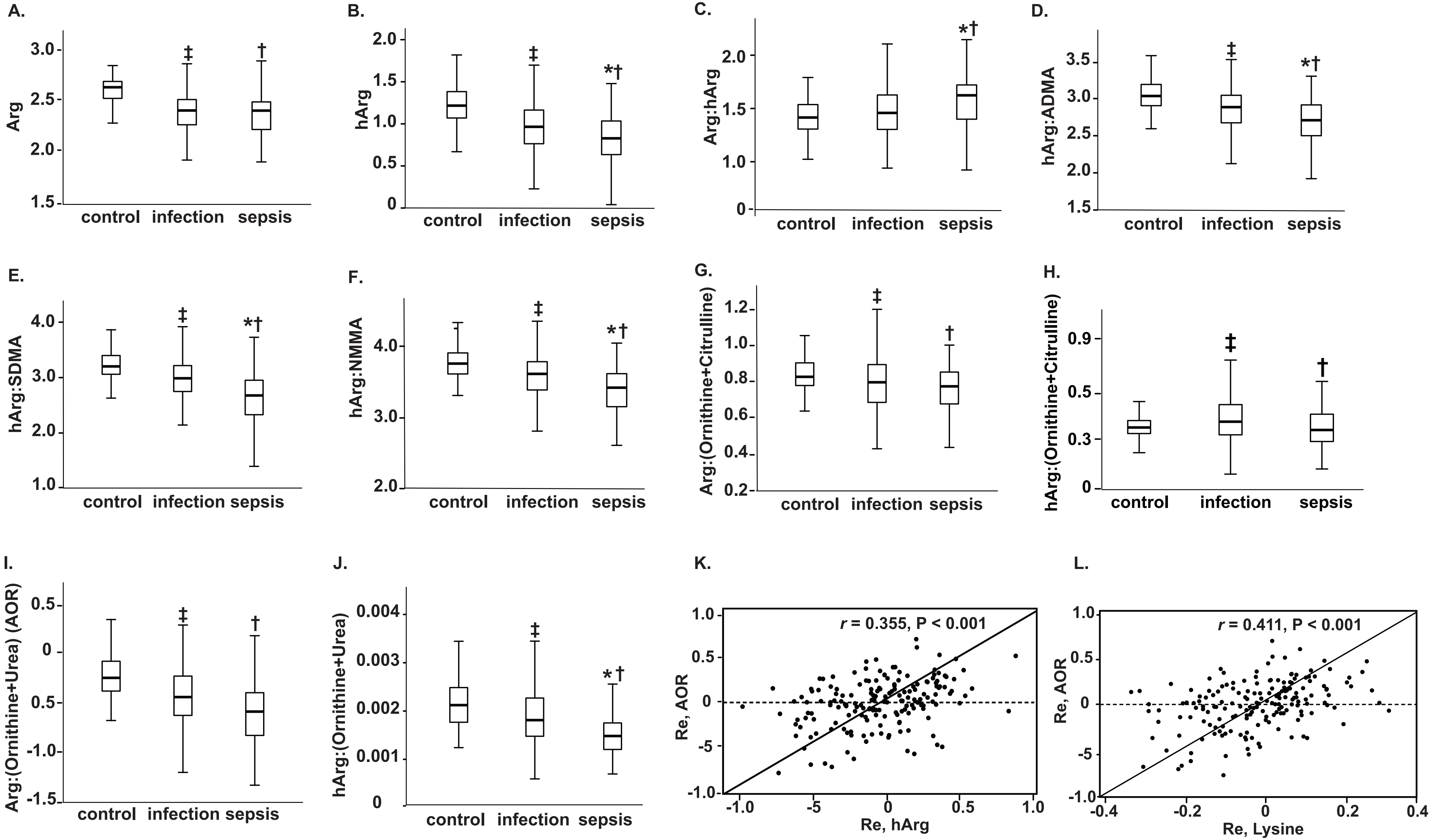

Supplemental Fig. 5 A-J. Patterns of plasma metabolites in control (n=82), infection (n=255), and sepsis (n=128) groups. Data are log transformed. Boxplots show median and IQR for each metabolite, and the whiskers are 10th and 90th percentiles. Difference between groups were compared using non-parametric Kruskal–Wallis test or Mann-Whitney U test, as appropriate. P-value with Bonferroni correction was adjusted for multiple tests. ‡ $P < 0.001$ for differences between control and infection, *$P < 0.001$ for differences between sepsis and control, †$P < 0.001$ for differences between sepsis and infection. K-L. Partial correlation plots between hArg, lysine and arginine-to-ornithine-urea ratio (AOR), adjusted for age, creatinine, and co-morbidities. Unstandardised residual (Re) indicates the Pearson correlation residuals between K. AOR and hArg, L. AOR and lysine, respectively. (hArg) homoarginine, (SDMA) Symmetric dimethylarginine, (ADMA) asymmetric dimethylarginine and (NNMA) NG-monomethyl-L-arginine. Nitric oxide synthase (NOS). (hArg:ADMA) plasma hArg/ADMA ratio, (hArg:SDMA) plasma hArg/SDMA ratio, and (hArg:NMMA) plasma hArg/NMMA ratio.

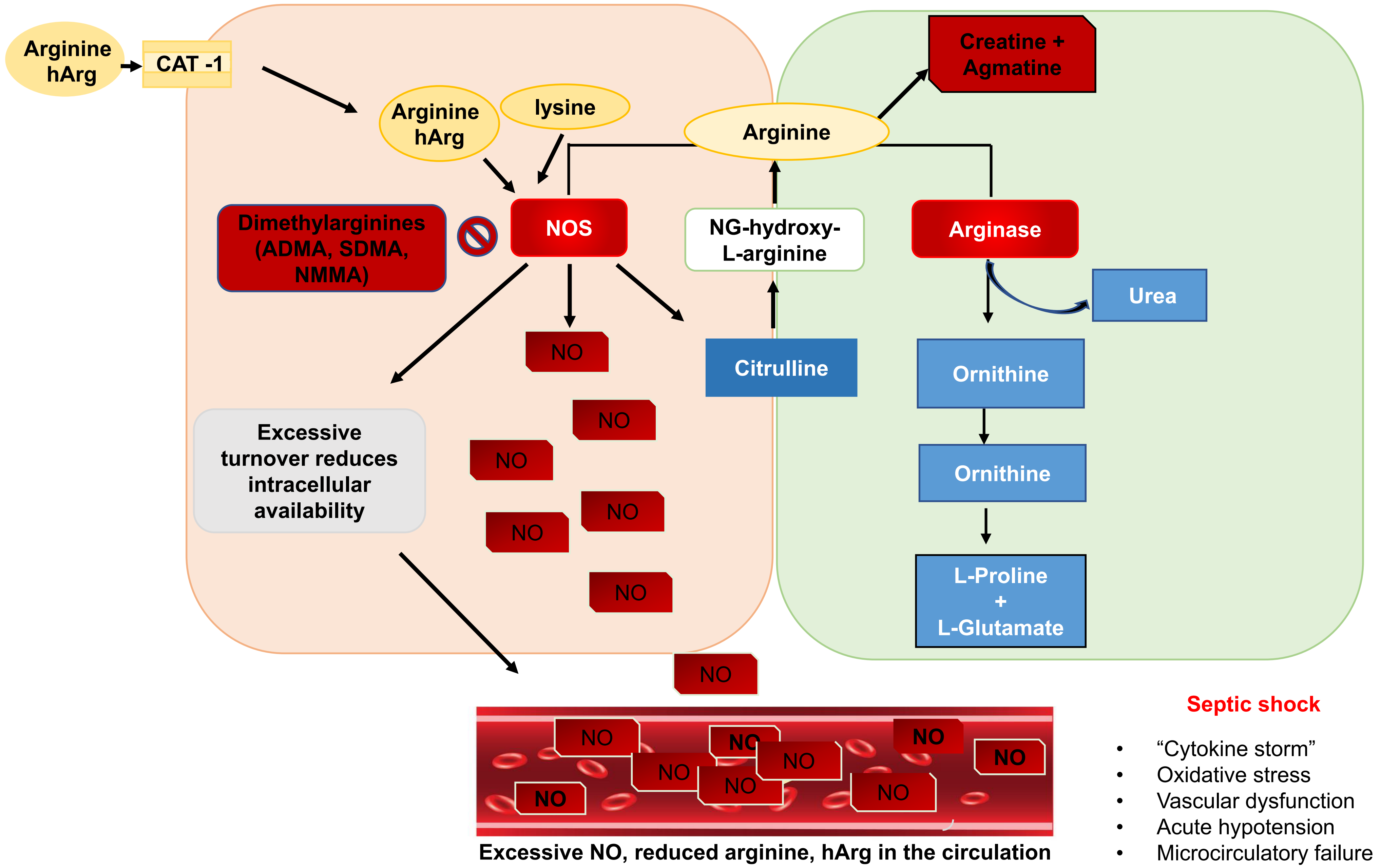

Supplemental Fig. 6 Arginine and NO metabolism in sepsis. Dysregulated arginine and nitric oxide metabolism and excessive NO production, leading to massive cytokines secretion, oxidative stress, vascular endothelial dysfunction, consequently microcirculatory failure in septic shock.
